# Development of an Exercise Intervention Program During Dialysis for Patients on Maintenance Hemodialysis Based on the Health Belief Model

**DOI:** 10.1155/jonm/5514298

**Published:** 2026-07-10

**Authors:** Xianjuan Cheng, Heng Dai, Yuanchun Guan, Chunling Xia, Hailong Hou, He Li, Hangfei Qu, Shiqi Xiao

**Affiliations:** ^1^ Department of Nursing (Department of Nephrology), Shengjing Hospital of China Medical University, Shenyang, Liaoning, China, cmu.edu.cn; ^2^ Department of Nursing (Department of Obstetrics and Gynecology), Shengjing Hospital of China Medical University, Shenyang, Liaoning, China, cmu.edu.cn; ^3^ Department of Nursing, Shengjing Hospital of China Medical University, Shenyang, Liaoning, China, cmu.edu.cn; ^4^ Department of Nursing (Department of Pediatric Hematology), Shengjing Hospital of China Medical University, Shenyang, Liaoning, China, cmu.edu.cn; ^5^ Inpatient Department, Shengjing Hospital of China Medical University, Shenyang, Liaoning, China, cmu.edu.cn

**Keywords:** evidence summary, exercise intervention, maintenance hemodialysis, program construction

## Abstract

**Objective:**

This study aims to develop an exercise intervention program for maintenance hemodialysis patients during dialysis based on the Health Belief Model, providing a scientific reference for the implementation and clinical guidance of intradialytic exercise interventions.

**Methods:**

Guided by the evidence pyramid model, a comprehensive computer‐based search was conducted to identify literature related to intradialytic exercise in maintenance hemodialysis patients. The search covered publications from database inception to October 2023. The quality of the included studies was assessed using a standardized literature quality evaluation system. Based on the Health Belief Model, the available evidence was synthesized to formulate a preliminary exercise intervention program, which was subsequently refined through expert consultation to establish the final intervention program for maintenance hemodialysis patients during dialysis.

**Results:**

A total of 15 eligible studies were included, consisting of 1 clinical decision, 2 guidelines, 3 expert consensus statements, and 9 systematic reviews. The evidence was summarized into five key domains: the necessity of exercise, pre‐exercise preparation, exercise prescription, exercise monitoring, and exercise support. Following expert consultation, the final exercise intervention program was established, comprising 5 first‐level items, 15 second‐level items, and 44 third‐level items.

**Conclusion:**

The evidence‐based summary of intradialytic exercise for maintenance hemodialysis patients, developed within the Health Belief Model framework, demonstrates strong scientific validity and practical applicability. This program provides a reliable foundation for future implementation and clinical guidance of exercise interventions during dialysis, while also promoting patients’ voluntary participation in healthy exercise behaviors and improving exercise adherence. Nursing managers can use this exercise intervention as a foundation to develop unit‐specific standardized protocols and incentive systems for intradialytic exercise. They should integrate exercise intervention into nurses’ routine duties and organize targeted training programs. Additionally, establishing quality monitoring indicators will help promote the sustainable implementation of intradialytic exercise.

## 1. Introduction

Maintenance hemodialysis (MHD) is an effective renal replacement therapy for patients with end‐stage renal disease (ESRD) caused by various primary and secondary etiologies, particularly those progressing to uremia. By improving quality of life and prolonging survival, MHD has become one of the principal clinical approaches for sustaining patients with ESRD [1]. With the global aging population and the increasing prevalence of chronic conditions such as hypertension and diabetes, the number of patients requiring hemodialysis continues to rise worldwide each year [2]. In China, the prevalence of kidney disease requiring dialysis has steadily increased annually [3], and the country currently has the largest dialysis population globally [4]. According to data from the China Renal Data System (CNRDS), the number of hemodialysis patients in China reached 1 million by the end of 2023, the highest recorded to date. In recent years, continuous advances in hemodialysis technology have significantly prolonged the survival of MHD patients. However, these improvements have also been accompanied by several emerging challenges, including reduced physical function, anxiety and depression, sleep disorders, and impaired quality of life [5–8]. These complications not only affect patient well‐being but also impose substantial economic and caregiving burdens on families and society [9–12]. As healthcare priorities increasingly shift toward improving overall quality of life, treatment goals for MHD patients have evolved beyond merely prolonging survival to promoting comprehensive physical and psychological health [13, 14]. Over the past decade, numerous domestic and international studies have demonstrated that exercise training during dialysis can improve dialysis adequacy, alleviate microinflammatory responses, enhance physical function, improve psychological status, and ultimately increase the daily quality of life of MHD patients [5, 15–17]. Among these benefits, dialysis adequacy is considered an independent predictor of survival outcomes in patients with ESRD. Rochmawati et al. [18] reported that dialysis adequacy independently predicts mortality risk. Furthermore, the 2015 Kidney Disease Outcomes Quality Initiative (KDOQI) Clinical Practice Guideline for Hemodialysis Adequacy [19] issued by the National Kidney Foundation recommends a target single‐pool Kt/V (spKt/V) of 1.4 per hemodialysis session, with a minimum delivered spKt/V of 1.2. Previous studies have also shown that exercise interventions during dialysis sessions produce more favorable Kt/V outcomes than exercise before dialysis or on nondialysis days [20]. However, existing research on intradialytic exercise interventions for MHD patients, both in China and internationally, has primarily focused on the effects of isolated factors on patient‐related outcomes [21, 22]. Evidence‐based summaries and standardized guidance for exercise management programs remain limited [23], with related evidence syntheses emerging only within the past 2 years. Moreover, the current exercise status of MHD patients remains suboptimal [24], highlighting the need to further improve both exercise capacity and adherence in this population.

## 2. Materials and Methods

### 2.1. Construction of the First Draft of the Program

#### 2.1.1. Theoretical Foundation

**FIGURE 1:**
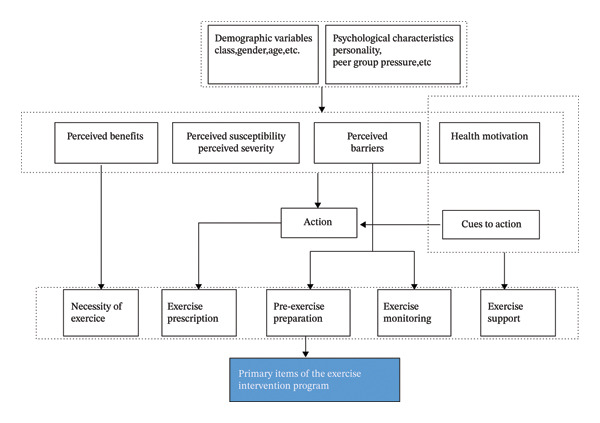
Theoretical framework of the health belief model in this study.

Psychological factors influencing exercise participation among MHD patients include their understanding of the benefits and barriers of exercise, negative emotions such as fear and anxiety, and levels of exercise self‐efficacy. To address these issues, several researchers have recommended applying appropriate theoretical frameworks to support patients in initiating and maintaining exercise behaviors [25]. The Health Belief Model (HBM) explains the mechanisms underlying health behavior change from a psychosocial perspective. Through its core components, including perceived benefits, perceived barriers, and behavioral cues, the model can effectively guide patients in overcoming obstacles to exercise and developing proactive participation in intradialytic exercise activities. Based on this theoretical framework, this study applied the HBM to the MHD patient population to develop a systematic exercise intervention program during dialysis sessions, providing a scientific basis for exercise interventions in this patient population. The theoretical framework adopted in this study is presented in Figure 1.

#### 2.1.2. Literature Review

This study was conducted and reported in accordance with the Preferred Reporting Items for Systematic Reviews and Meta‐Analyses (PRISMA) statement. A literature analysis approach was adopted to retrieve relevant evidence on exercise interventions during MHD, using the “5S” evidence model. Two master’s students, trained in evidence‐based nursing, independently conducted the literature search and evaluated the methodological quality of the included studies using internationally recognized appraisal tools. The Appraisal of Guidelines for Research and Evaluation II (AGREE II) instrument was used to assess guidelines, the Assessment of Multiple Systematic Reviews (AMSTAR) tool was applied to systematic reviews, and the evaluation criteria of the Joanna Briggs Institute (JBI) Evidence‐Based Health Care Center (2016 Version) [26] were used for other types of literature. Any disagreements regarding study inclusion, exclusion, or quality assessment were resolved through discussion with a third researcher specializing in evidence‐based nursing. The synthesized evidence was subsequently used to guide the development of an intradialytic exercise intervention protocol and to formulate a preliminary exercise program for MHD patients. The initial program primarily covered five aspects: the necessity of exercise, pre‐exercise preparation, exercise prescription, exercise monitoring, and exercise support.

#### 2.1.3. Search Strategy

A systematic search was conducted across multiple databases and professional association websites, including BMJ Best Practice, UpToDate, the Guidelines International Network (GIN), the World Health Organization (WHO), the JBI Evidence‐Based Healthcare Database, the Cochrane Library, PubMed, Yimaitong, China National Knowledge Infrastructure (CNKI), the Chinese Biomedical Literature Database (CBM), VIP Database, and Wanfang Database. Relevant professional association websites were searched, including those of The Renal Association (RA), the British Association of Sport and Exercise Sciences (BASES), the Chinese Society of Nephrology, and the American College of Sports Medicine (ACSM). The literature search was performed using a combination of Medical Subject Headings (MeSH) and free‐text terms. The primary search terms included “dialysis,” “hemodialysis,” “hemodialysis,” “renal dialysis,” “exercise,” “physical activities,” “training,” “guideline,” “expert consensus,” “systematic review,” “meta‐analysis,” “practice guidelines as topic,” and “summary of evidence.” The retrieval period covered all publications from the establishment of each database through October 2023. Taking the PubMed database as an example, the search query is as follows:

 #1 (“renal dialysis” [MeSH Terms] OR “haemodialysis” [Title/Abstract] OR “dialysis” [Title/Abstract] OR “hemodialysis” [Title/Abstract])
 #2 (“exercise” [MeSH Terms] OR “physical activities” [Title/Abstract] OR “training” [Title/Abstract])
 #3 “guideline” [Publication Type] OR “systematic review” [Publication Type] OR “meta‐analysis” [Publication Type] OR “practice guidelines as topic” [Publication Type]OR “summary of evidence” [Publication Type] OR “expert consensus” [Publication Type]
 #4 #1 AND #2 AND #3

#### 2.1.4. Literature Inclusion and Exclusion Criteria

##### 2.1.4.1. Inclusion Criteria

The study population consisted of MHD patients who had undergone dialysis treatment for at least 3 months. The intervention measures included exercise‐based interventions implemented during dialysis sessions. Outcome indicators included dialysis adequacy, anxiety and depression, and quality of life. Eligible literature types comprised clinical decisions, guidelines, expert consensus statements, systematic reviews, and evidence summaries.

##### 2.1.4.2. Exclusion Criteria

Studies were excluded if they were duplicate publications, published in languages other than Chinese or English, lacked sufficient information or full‐text accessibility, were conference abstracts, or failed to meet the required methodological quality assessment standards.

#### 2.1.5. Literature Quality Evaluation Standards and Process

All assessors involved in this study underwent standardized training in methods of literature quality appraisal to ensure consistency in the evaluation process. Clinical guidelines were assessed using the AGREE II instrument, with four researchers independently evaluating six domains, including scope and purpose, stakeholder involvement, rigor of development, and clarity of presentation. Final evaluation results were determined through group discussion and consensus. Systematic reviews were evaluated using the AMSTAR tool, in which two reviewers independently assessed 11 items and classified each as “yes,” “no,” “unclear,” or “not applicable.” For expert consensus statements and clinical decision literature, the corresponding appraisal tools recommended by the JBI Evidence‐Based Health Care Center were applied, with two reviewers independently evaluating each item. Throughout the assessment process, all researchers strictly followed standardized evaluation procedures. Any disagreements between reviewers were resolved through discussion, and when necessary, a third evaluator participated in the final decision‐making process. In cases where evidence conclusions were inconsistent, priority was given to high‐quality evidence and the most recently published studies to ensure the scientific validity and reliability of the findings.

#### 2.1.6. Literature Inclusion Status

**FIGURE 2:**
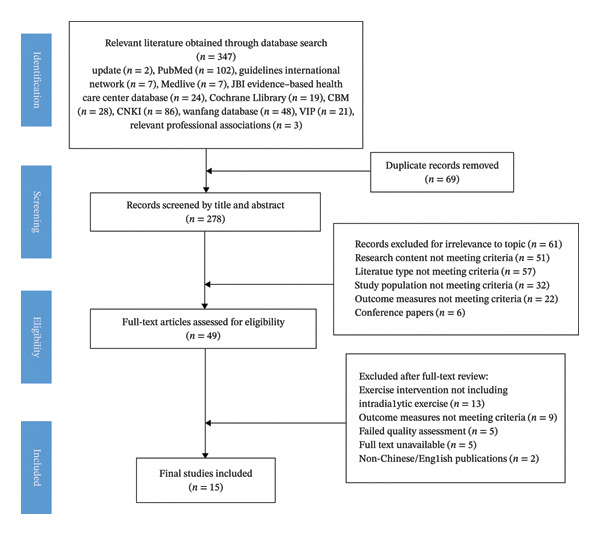
PRISMA diagram.

A total of 347 articles were initially identified in this study, of which 69 duplicate records were removed. Following literature screening and methodological quality assessment, 15 eligible studies were ultimately included, comprising 1 clinical decision, 2 guidelines, 3 expert consensus statements, and 9 systematic reviews. The literature selection process is illustrated in Figure 2, while the general characteristics of the included studies are summarized in Table 1.

**TABLE 1:** Literature information extraction table.

Serial number	Included literature	Publication year	Literature source	Literature type	Literature topic
1	Miller et al. [27]	2020	UpToDate	Clinical Decision	Uremic Myopathy and Deconditioning in Chronic Kidney Disease Patients
2	Ashby et al. [28]	2019	The Renal Association	Guideline	Clinical Practice Guidelines for Hemodialysis
3	Chinese Association of Rehabilitation Medicine [29]	2021	Yimaitong Guideline Network	Expert Consensus	Expert Consensus on the Construction of a Rehabilitation System for Hemodialysis Centers
4	Zhang et al. [30]	2021	CNKI	Expert Consensus	Expert Consensus on Rehabilitation Therapy for Adult Hemodialysis Patients in China
5	Baker et al. [31]	2021	The Renal Association	Guideline	Clinical Practice Guidelines: Exercise and Lifestyle for Chronic Kidney Disease Patients
6	Koufaki et al. [32]	2015	BASES	Expert Consensus	BASES Expert Consensus: Exercise Therapy for Chronic Kidney Disease Patients
7	Huang et al. [33]	2019	PubMed	Systematic Review	Systematic Review and Meta‐Analysis of Exercise Training and Outcomes in Maintenance Hemodialysis Patients
8	Hargrove et al. [34]	2021	PubMed	Systematic Review	The Impact of Aerobic Training on Dialysis‐Related Symptoms in Maintenance Hemodialysis Patients: A Systematic Review and Meta‐Analysis of Clinical Trials
9	Cai et al. [35]	2022	PubMed	Systematic Review	Systematic Review and Meta‐Analysis of the Efficacy of Aerobic Exercise Combined with Resistance Training in Maintenance Hemodialysis Patients
10	Andrade et al. [36]	2019	PubMed	Systematic Review	Literature Analysis of Intradialytic Exercise Programs for Maintenance Hemodialysis Patients
11	Chen et al. [37]	2022	CNKI	Systematic Review	Systematic Review | Meta‐Analysis of the Impact of Exercise Intervention on Physical Function in Maintenance Hemodialysis Patients
12	Xintao et al. [38]	2021	CNKI	Systematic Review	Meta‐Analysis of the Impact of Intradialytic Exercise on Exercise Capacity and Dialysis Efficiency in Maintenance Hemodialysis Patients
13	Wang et al. [39]	2021	CNKI	Systematic Review	Meta‐Analysis of the Effect of Intradialytic Exercise on Improving the Quality of Life in Maintenance Hemodialysis Patients
14	Wang et al. [15]	2019	Wanfang	Systematic Review	Meta‐Analysis of the Impact of Intradialytic Aerobic Exercise on Dialysis Adequacy, Microinflammatory State, and Albumin Levels in Patients
15	Bernier‐Jean et al. [40]	2022	Cochrane Library	Systematic Review	Exercise training for adults undergoing maintenance dialysis

#### 2.1.7. Quality Assessment Results of Included Literature

The quality assessment results of the two included guidelines showed that one guideline achieved standardized scores of ≥ 60% across all six evaluation domains. In comparison, the other guideline achieved standardized scores of ≥ 60% in five domains and a score of 30%–60% in one domain. Among the nine included systematic reviews, quality assessment revealed that seven studies were rated as “No” for the criterion, “Were the inclusion criteria based on publication status, such as grey literature?” In addition, three systematic reviews were rated as “No” for the criterion, “Was the relevant conflict of interest stated?” All remaining assessment items across the systematic reviews were rated as “Yes.” The quality appraisal results for the clinical decisions and expert consensus documents were all rated as “Yes.” Detailed results of the literature quality assessment are presented in Tables 2, 3, and 4.

**TABLE 2:** Guideline appraisal results.

Included guideline	Standardized percentage for each domain						Number of domains with score ≥ 60%	Number of domains with score < 60% and ≥ 30%	Number of domains with score < 30%	Recommendation grade
	Scope and Purpose	Stakeholder Involvement	Rigor of Development	Clarity of Presentation	Applicability	Editorial Independence				
Ashby et al. [28]	98.61	95.83	93.06	78.13	41.67	70.83	5	1	0	B
Baker et al. [31]	98.61	97.22	98.61	90.63	93.75	97.92	6	0	0	A

**TABLE 3:** Quality appraisal results of systematic reviews.

Evaluation item	Appraisal result								
	Huang et al. [33]	Hargrove et al. [34]	Cai et al. [35]	Andrade et al. [36]	Chen et al. [37]	Xintao et al. [38]	Wang et al. [39]	Wang et al. [15]	Bernier‐Jean et al. [40]
1. Was an a priori design provided?	Yes	Yes	Yes	Yes	Yes	Yes	Yes	Yes	Yes
2. Was there duplicate study selection and data extraction?	Yes	Yes	Yes	Yes	Yes	Yes	Yes	Yes	Yes
3. Was a comprehensive literature search performed?	Yes	Yes	Yes	Yes	Yes	Yes	Yes	Yes	Yes
4. Did the inclusion criteria consider publication status, such as grey literature?	No	Yes	No	No	No	No	No	No	Yes
5. Was a list of included and excluded studies provided?	Yes	Yes	Yes	Yes	Yes	Yes	Yes	Yes	Yes
6. Were the characteristics of the included studies provided?	Yes	Yes	Yes	Yes	Yes	Yes	Yes	Yes	Yes
7. Was the scientific quality of the included studies assessed and documented?	Yes	Yes	Yes	Yes	Yes	Yes	Yes	Yes	Yes
8. Was the scientific quality of the included studies used appropriately in formulating conclusions?	Yes	Yes	Yes	Yes	Yes	Yes	Yes	Yes	Yes
9. Were the methods used to combine the findings of studies appropriate?	Yes	Yes	Yes	Yes	Yes	Yes	Yes	Yes	Yes
10. Was the likelihood of publication bias assessed?	Yes	Yes	Yes	Yes	Yes	Yes	Yes	Yes	Yes
11. Was the conflict of interest stated?	Yes	No	Yes	Yes	Yes	Yes	No	No	Yes

**TABLE 4:** Quality appraisal results of clinical decision support and expert consensus documents.

Evaluation item	Appraisal result			
	Miller [27]	Chinese rehabilitation medicine association [29]	Zhang et al. [30]	Koufaki et al. [32]
1. Is the source of the viewpoints clearly stated?	Yes	Yes	Yes	Yes
2. Do the viewpoints originate from influential experts in the field?	Yes	Yes	Yes	Yes
3. Are the viewpoints patient‐centered?	Yes	Yes	Yes	Yes
4. Are the conclusions logically presented and based on analytical results?	Yes	Yes	Yes	Yes
5. Is existing literature referenced?	Yes	Yes	Yes	Yes
6. Do the presented viewpoints have any discrepancies with the existing literature?	Yes	Yes	Yes	Yes

#### 2.1.8. Description and Summary of Evidence

Based on the HBM, relevant evidence was extracted from the 15 included studies. Following evidence classification and synthesis, a preliminary exercise program for MHD patients was developed. The initial draft consisted of 5 first‐level categories, including the necessity of exercise, pre‐exercise preparation, exercise prescription, exercise monitoring, and exercise support, along with 14 second‐level categories and 40 third‐level categories.

### 2.2. Delphi Method

#### 2.2.1. Establishment of the Research Team

This study adopted the Delphi expert consultation method. It established a research team of 7 members, including the principal researcher, the graduate supervisor, a nephrology clinical expert, the head nurse of the hemodialysis center, and 3 graduate students. Regarding professional qualifications, the team consisted of two senior‐level professionals, one intermediate‐level professional, and four junior‐level professionals. The main responsibilities of the research team included designing and refining the consultation questionnaire, selecting appropriate experts for consultation, and organizing, summarizing, and analyzing the feedback and recommendations provided by the experts.

#### 2.2.2. Design of the Consultation Questionnaire

Based on the preliminary exercise intervention program for MHD patients, a questionnaire for the first round of expert consultation was initially developed. The questionnaire mainly consisted of four sections: (1) an introduction to the research background and objectives to help experts gain a comprehensive understanding of the study content; (2) a basic information survey to collect data regarding the experts’ professional background and work experience; (3) an expert consultation form on the exercise intervention program, including scoring and evaluation of the first‐level, second‐level, and third‐level items; (4) a self‐assessment questionnaire evaluating the experts’ familiarity (Cs) and judgment basis (Ca), which was used to assess expert authority. Following the first round of consultation, the research team reviewed and discussed the experts’ feedback and recommendations, revised the questionnaire accordingly, and developed the second‐round consultation questionnaire for further expert evaluation.

#### 2.2.3. Selection of Consultation Experts

Following discussion within the research team, the expert selection criteria for this study were established as follows: (1) experts were required to be engaged in medical, clinical rehabilitation, or nursing practice in comprehensive tertiary hospitals and possess at least 10 years of professional experience; (2) experts were required to hold intermediate or higher professional technical titles; (3) the minimum academic qualification was a bachelor’s degree, with preference given to individuals holding master’s degrees or higher; and (4) experts were expected to have substantial clinical experience and strong research expertise in relevant fields, enabling them to provide comprehensive and professional guidance for the study. A total of 12 experts participated in and completed both rounds of consultation. The composition of the expert panel remained consistent throughout the consultation process. Detailed information regarding the experts is presented in Table 5.

**TABLE 5:** Statistical results of basic information of consultation experts.

Item	Number	Percentage (%)
Age (years)		
30–40	2	16.67
41–50	8	66.67
≥ 51	2	16.67
Gender		
Male	3	25
Female	9	75
Education		
Bachelor’s degree	7	58.33
Master’s degree or higher	5	41.67
Professional Title		
Intermediate	10	83.33
Senior	2	16.67
Years of Work Experience		
10–20	8	66.67
21–30	4	33.33

#### 2.2.4. Expert Consultation Process

In this study, expert consultation questionnaires were distributed and collected via both paper‐based forms and email. Experts were asked to complete and return the questionnaires within 2 weeks of receipt. They were required to evaluate the importance and operability of each item and provide recommendations for modification, deletion, or addition in the “revision comments” section. Based on the experts’ evaluation results, items with mean importance and operability scores ≥ 3.5 and a coefficient of variation (CV) < 0.25 [41] were retained. The research team then revised the relevant items according to the experts’ feedback and developed the subsequent round of the consultation questionnaire for further evaluation. The expert consultation questionnaires used in both rounds are presented in Appendices 1 and 2.

### 2.3. Statistical Methods

Data processing and statistical analysis in this study were performed using SPSS 19.0 Software. Quantitative variables were expressed as mean ± standard deviation, while categorical variables were presented as frequency and percentage. The level of expert engagement was evaluated based on the questionnaire response rate. Expert authority was assessed using the authority coefficient (Cr), calculated as the arithmetic mean of the expert’s judgment basis (Ca) and familiarity (Cs), as follows: Cr = (Ca + Cs)/2. A Cr value ≥ 0.7 was considered indicative of a high level of expert authority. The degree of consensus among experts was evaluated using the CV and Kendall’s coefficient of concordance (W). A lower CV value indicated reduced variability and greater consistency in expert opinions. In comparison, a higher W value (ranging from 0 to 1) reflected stronger agreement among experts on the proposed intervention items, suggesting greater coordination and consensus within the expert panel.

## 3. Results

### 3.1. Expert Consultation Results

#### 3.1.1. Expert Response Rate and Authority

In the first round of expert consultation, a total of 15 questionnaires were distributed: 6 via email and 9 in paper form. Of these, 12 questionnaires were returned and considered valid, yielding an effective response rate of 80%. In the second round, 12 questionnaires were distributed: 3 via email and 9 paper‐based. All were returned with complete responses, resulting in a 100% response rate. The authority coefficients (Cr) for the first and second rounds were 0.830 and 0.829, respectively. Both values exceeded the accepted threshold of 0.7, indicating a high level of authority.

#### 3.1.2. Coordination Degree of Expert Opinions

Following the second round of consultation, the mean scores for item importance and operability ranged from 4.00 to 4.92 and 4.08 to 4.92, respectively. The corresponding coefficients of variation ranged from 0.06 to 0.15 for importance and from 0.06 to 0.18 for operability, all of which were below the 0.25 threshold, indicating a trend toward consensus among the experts. The Kendall’s coefficients of concordance (W) for both rounds were 0.24, and the associated significance tests yielded *p* values < 0.05, demonstrating good coordination among the experts and supporting the reliability of the developed intervention program. As satisfactory consensus had been achieved, no additional rounds of consultation were conducted. Detailed results of the two rounds of Delphi consultation are presented in Tables 6 and 7.

**TABLE 6:** First‐round expert consultation results of an exercise intervention program for maintenance hemodialysis patients based on the health belief model.

Subjects	Importance (*n* = 12)			Operationalizability (*n* = 12)		
	Mean	SD	CV	Mean	SD	CV
1 Necessity of Exercise	4.83	0.39	0.08	4.67	0.49	0.11
1.1 Exercise Safety	4.83	0.39	0.08	4.92	0.29	0.06
1.1.1 Exercise during dialysis is safe, and it is recommended that all treatment institutions provide exercise during dialysis to assist in treatment.	4.17	0.39	0.09	4.5	0.67	0.15
1.2 Exercise Benefits	4.83	0.39	0.08	4.92	0.29	0.06
1.2.1 Exercising for at least 60 min three times a week during dialysis can improve the survival rate of dialysis patients.	4.42	0.52	0.12	4.17	0.72	0.17
1.2.2 Moderate‐intensity resistance and aerobic exercise during dialysis can improve dialysis adequacy.	4.33	0.49	0.11	4.33	0.78	0.18
1.2.3 Exercise during dialysis can significantly improve microinflammatory status, serum albumin levels, fatigue, and sleep conditions in dialysis patients.	4.33	0.49	0.11	4.67	0.65	0.14
1.2.4 Exercise during dialysis can improve physical function, quality of life, cardiopulmonary function, and blood pressure levels in dialysis patients.	4.08	0.52	0.13	4.33	0.49	0.11
1.2.5 Exercise during dialysis can alleviate psychological disorders such as depression and anxiety in patients.	4.25	0.62	0.15	4.25	0.62	0.15
1.2.6 It is worth emphasizing that even a slight increase in physical activity levels may be beneficial.	4.08	0.29	0.07	4.33	0.78	0.18
2 Pre‐Exercise Preparation	4.83	0.39	0.08	4.67	0.49	0.11
2.1 Researcher Preparation	4.42	0.52	0.12	4.25	0.45	0.11
2.1.1 It is recommended that professional medical staff develop the exercise plan and implement it accordingly.	4.92	0.29	0.06	4.83	0.39	0.08
2.1.2 Researchers should enhance their knowledge of relevant expertise, educate dialysis patients and their families about the implementation methods, safety monitoring, and outcome evaluation, and fully recognize its clinical benefits.	4.5	0.52	0.12	4.5	0.52	0.12
2.2 Patient Preparation	4.33	0.65	0.15	4.25	0.75	0.18
2.2.1 Patients should be informed of the benefits and risks before exercise, and informed consent should be obtained from the patient/family, with a signed consent form.	4.5	0.52	0.12	4.58	0.52	0.11
2.2.2 Ensure the patency of vascular access, as it is the lifeline for dialysis patients and a necessary condition for adequate dialysis.	4	0.43	0.11	4.08	0.52	0.13
2.3 Exercise Safety Assessment	4.42	0.67	0.15	4.33	0.78	0.18
2.3.1 Clinical Condition Assessment: ① Medical history assessment: Symptoms, comorbidities (especially cardiovascular diseases, bone and joint abnormalities, etc.), medication history, lifestyle habits, exercise habits, family history, etc. ② Physical examination ③ Laboratory tests.	4.75	0.45	0.10	4.67	0.49	0.11
2.3.2 Exercise Capacity Assessment: Measurement of Maximal Oxygen Uptake (VO_2_max).	4.5	0.52	0.12	2.83	0.72	0.25
2.4 Exercise Contraindications	4.58	0.52	0.11	4.75	0.45	0.10
2.4.1 Abnormal Blood Pressure: Severe hypertension (blood pressure exceeding 180/110 mmHg) or hypotension (blood pressure below 90/60 mmHg).	4.75	0.45	0.10	4.5	0.52	0.12
2.4.2 Cardiopulmonary Diseases: Severe heart failure, arrhythmias, unstable angina, severe pericardial effusion, valvular stenosis, hypertrophic cardiomyopathy, aortic dissection, uncontrolled pulmonary hypertension (mean pulmonary artery pressure > 55 mmHg).	4.25	0.45	0.11	4.33	0.49	0.11
2.4.3 Acute Clinical Events: Acute systemic inflammatory diseases or fever, acute phase of cardiovascular or cerebrovascular diseases, acute phase of traumatic injuries, etc.	4.08	0.52	0.13	4.33	0.49	0.11
2.4.4 New‐Onset Deep Vein Thrombosis Symptoms: Exercise should be postponed or stopped if symptoms such as abnormal redness, swelling, or pain in the calf occur.	4.17	0.39	0.09	4.5	0.52	0.12
2.4.5 Patient Inability to Cooperate with Exercise: If the patient is unable to cooperate with the exercise program.	4.83	0.39	0.08	4.75	0.45	0.10
3 Exercise Prescription (FITT^a^ Principle)	4.83	0.39	0.08	4.92	0.29	0.06
3.1 Exercise Frequency	4.42	0.52	0.12	4.75	0.45	0.10
3.1.1 Exercise frequency should be 3 times per week.	4.83	0.39	0.08	2.5	0.80	0.32
3.2 Exercise Intensity	4.42	0.67	0.15	4.92	0.29	0.06
3.2.1 Start with low‐intensity exercise and gradually progress to moderate‐intensity exercise.	4.08	0.29	0.07	4.5	0.52	0.12
3.2.2 It is recommended to use the Rating of Perceived Exertion (RPE)^b^ scale to determine exercise intensity. This scale is simple and practical. The exercise intensity is generally set at 12–14 points (the patient feels slightly tired but can still comfortably converse without significant strain). This ensures the patient achieves safe and effective exercise outcomes without losing exercise adherence.	4.83	0.39	0.08	4.58	0.52	0.11
3.3 Exercise Duration	4.25	0.75	0.18	4.92	0.29	0.06
3.3.1 The optimal time for exercise is between 30 min and 2 h after starting dialysis.	4.17	0.39	0.09	4.17	0.39	0.09
3.3.2 The target exercise duration is 30–60 min (including warm‐up and cool‐down). At least 30 min of exercise should be completed, either in a single session or divided into multiple sessions based on the patient’s condition.	4.75	0.45	0.10	4.75	0.45	0.10
3.3.3 The exercise program should last for at least 3 months.	4.67	0.65	0.14	4.33	0.65	0.15
3.4 Exercise Type	4.58	0.52	0.11	4.33	0.65	0.15
3.4.1 A single exercise session should include warm‐up, exercise training, and cool‐down. Warm‐up: At least 5–10 min of low to moderate‐intensity aerobic and muscle endurance exercises. Exercise phase: At least 20–60 min, including aerobic exercise, resistance exercise, and flexibility exercises. Cool‐down: At least 5–10 min of low to moderate‐intensity aerobic and muscle endurance exercises.	4.33	0.49	0.11	4.08	0.52	0.13
3.4.2 During dialysis, a combination of aerobic and resistance exercises, resistance exercises alone, or aerobic exercises alone can improve dialysis adequacy. Probability ranking shows that a combination of aerobic and resistance exercises during dialysis has the best effect on improving dialysis adequacy.	4	0.43	0.11	4.5	0.52	0.12
3.4.3 It is recommended that dialysis centers use motorized bicycles with adjustable resistance for routine aerobic and resistance exercises, as this method is simple and effective. Alternatively, a combination of aerobic, resistance, and flexibility exercises in a supine position can be performed during dialysis.	4.67	0.49	0.11	4.75	0.45	0.10
3.4.4 Aerobic exercises during dialysis can include various movements of the nonarteriovenous fistula arm and both lower limbs, such as hand gripping, wrist rotation, elbow flexion and extension, and hip adduction, abduction, flexion, and extension.	4.5	0.67	0.15	4.58	0.52	0.11
3.4.5 Resistance exercises during dialysis can include lifting dumbbells with the nonfistula arm, elastic band training, progressive ankle weight training, resistance band exercises, knee extension exercises, and progressive supine leg lifts.	4.5	0.52	0.12	4.5	0.52	0.12
4 Exercise Monitoring	4.67	0.49	0.11	4.75	0.45	0.10
4.1 Monitoring Timing and Content	4.33	0.49	0.11	4.5	0.67	0.15
4.1.1 Monitoring should occur before, during, and after exercise, including vital signs and vascular access status.	4.58	0.52	0.11	4.42	0.52	0.12
4.1.2 Strengthen monitoring during dialysis and assess exercise intensity. Closely observe the patient for any discomfort during exercise and identify potential causes.	4.33	0.49	0.11	4.58	0.52	0.11
4.2 Exercise Termination Criteria	4.5	0.52	0.12	4.33	0.65	0.15
4.2.1 Persistent chest or back pain, palpitations, or chest tightness.	4.5	0.52	0.12	4.5	0.52	0.12
4.2.2 Severe shortness of breath or difficulty speaking.	4.42	0.52	0.12	4.25	0.45	0.11
4.2.3 Headache, dizziness, generalized weakness, visual disturbances, or profuse sweating.	4.67	0.49	0.11	4.42	0.52	0.12
4.2.4 Severe arrhythmias.	4.42	0.52	0.12	4.5	0.52	0.12
4.2.5 Exercise‐related muscle cramps or joint pain.	4.33	0.65	0.15	4.58	0.52	0.11
5 Exercise Support	4.92	0.29	0.06	4.83	0.39	0.08
5.1 Theoretical Support	4.83	0.39	0.08	4.25	0.75	0.18
5.1.1 Use appropriate theories (e.g., Health Belief Model, Theory of Planned Behavior) to help patients adopt and adhere to exercise plans.	4.58	0.52	0.11	4.67	0.49	0.11
5.2 Social Support	4.92	0.29	0.06	4.58	0.67	0.15
5.2.1 Educate patients about exercise during dialysis, including indications, contraindications, benefits, risks, specific exercise protocols, precautions, and how to manage potential discomfort.	4.83	0.39	0.08	4.75	0.45	0.10
5.2.2 Encourage patients to use diaries, informational manuals, or other visual tools to track exercise progress. Provide demonstration materials (e.g., manuals, videos) in advance.	4.83	0.39	0.08	4.75	0.45	0.10
5.2.3 Train dialysis center staff on emergency response protocols.	4.67	0.65	0.14	4.42	0.67	0.15

^a^FITT stands for Frequency, Intensity, Time, and Type.

^b^It is a subjective method used to measure the intensity of exercise or physical activity. You rate your current level of fatigue based on your own feelings.

**TABLE 7:** Second‐round expert consultation results of an exercise intervention program for maintenance hemodialysis patients based on the health belief model.

Subjects	Importance (*n* = 12)			Operationalizability (*n* = 12)		
	Mean	SD	CV	Mean	SD	CV
1 Necessity of Exercise	4.75	0.45	0.10	4.83	0.39	0.08
1.1 Exercise Safety	4.83	0.39	0.08	4.92	0.29	0.06
1.1.1 Exercise during dialysis is safe, and it is recommended that all treatment institutions provide exercise during dialysis to assist in treatment.	4.17	0.39	0.09	4.75	0.45	0.10
1.2 Exercise Benefits	4.83	0.39	0.08	4.83	0.39	0.08
1.2.1 Exercising for at least 60 min three times a week during dialysis can improve the survival rate of dialysis patients.	4.5	0.52	0.12	4.17	0.58	0.14
1.2.2 Moderate‐intensity resistance and aerobic exercise during dialysis can improve dialysis adequacy.	4.17	0.39	0.09	4.33	0.78	0.18
1.2.3 Exercise during dialysis can significantly improve microinflammatory status, serum albumin levels, fatigue, and sleep conditions in dialysis patients.	4.33	0.49	0.11	4.67	0.65	0.14
1.2.4 Exercise during dialysis can improve physical function, quality of life, cardiopulmonary function, and blood pressure levels in dialysis patients.	4.25	0.62	0.15	4.33	0.49	0.11
1.2.5 Exercise during dialysis can alleviate psychological disorders such as depression and anxiety in patients.	4.08	0.52	0.13	4.25	0.62	0.15
1.2.6 It is worth emphasizing that even a slight increase in physical activity levels may be beneficial.	4.08	0.29	0.07	4.67	0.49	0.11
2 Pre‐Exercise Preparation	4.92	0.29	0.06	4.75	0.45	0.10
2.1 Researcher Preparation	4.33	0.49	0.11	4.50	0.52	0.12
2.1.1 It is recommended that professional medical staff develop the exercise plan and implement it accordingly.	4.75	0.45	0.10	4.83	0.39	0.08
2.1.2 Researchers should enhance their knowledge of relevant expertise, educate dialysis patients and their families about the implementation methods, safety monitoring, and outcome evaluation, and fully recognize its clinical benefits.	4.42	0.52	0.12	4.50	0.52	0.12
2.2 Patient Preparation	4.33	0.65	0.15	4.50	0.52	0.12
2.2.1 Patients should be informed of the benefits and risks before exercise, and informed consent should be obtained from the patient/family, with a signed consent form.	4.75	0.45	0.10	4.75	0.45	0.10
2.2.2 Ensure the patency of vascular access, as it is the lifeline for dialysis patients and a necessary condition for adequate dialysis.	4.17	0.39	0.09	4.17	0.39	0.09
2.2.3 Ensure adequate dialysis. Clinical issues such as volume overload, metabolic acidosis, anemia, malnutrition, and electrolyte imbalances often increase the risks of exercise implementation.	4.83	0.39	0.08	4.92	0.29	0.06
2.3 Exercise Safety Assessment	4.33	0.65	0.15	4.33	0.49	0.11
2.3.1 Clinical Condition Assessment: ① Medical history assessment: Symptoms, comorbidities (especially cardiovascular diseases, bone and joint abnormalities, etc.), medication history, lifestyle habits, exercise habits, family history, etc. ② Physical examination ③ Laboratory tests.	4.67	0.49	0.11	4.83	0.39	0.08
2.3.2 Exercise Capacity Assessment: Since over 50% of MHD patients cannot complete maximal oxygen uptake (VO2max) testing, it is recommended to use simple physical function tests, such as the 6‐min walk test.	4.75	0.45	0.10	4.92	0.29	0.06
2.4 Exercise Contraindications	4.58	0.52	0.11	4.75	0.45	0.10
2.4.1 Abnormal Blood Pressure: Severe hypertension (blood pressure exceeding 180/110 mmHg) or hypotension (blood pressure below 90/60 mmHg).	4.83	0.39	0.08	4.25	0.45	0.11
2.4.2 Cardiopulmonary Diseases: Severe heart failure, arrhythmias, unstable angina, severe pericardial effusion, valvular stenosis, hypertrophic cardiomyopathy, aortic dissection, uncontrolled pulmonary hypertension (mean pulmonary artery pressure > 55 mmHg).	4.08	0.29	0.07	4.17	0.39	0.09
2.4.3 Acute Clinical Events: Acute systemic inflammatory diseases or fever, acute phase of cardiovascular or cerebrovascular diseases, acute phase of traumatic injuries, etc.	4.17	0.58	0.14	4.17	0.39	0.09
2.4.4 New‐Onset Deep Vein Thrombosis Symptoms: Exercise should be postponed or stopped if symptoms such as abnormal redness, swelling, or pain in the calf occur.	4.42	0.52	0.12	4.50	0.52	0.12
2.4.5 Patient Inability to Cooperate with Exercise: If the patient is unable to cooperate with the exercise program.	4.67	0.65	0.14	4.75	0.45	0.10
3 Exercise Prescription (FITT Principle)	4.92	0.29	0.06	4.83	0.39	0.08
3.1 Exercise Frequency	4.42	0.52	0.12	4.75	0.45	0.10
3.1.1 Exercise frequency should be 2–3 times per week, based on the patient’s dialysis schedule.	4.83	0.39	0.08	4.83	0.39	0.08
3.2 Exercise Intensity	4.33	0.65	0.15	4.92	0.29	0.06
3.2.1 Start with low‐intensity exercise and gradually progress to moderate‐intensity exercise.	4.08	0.29	0.07	4.50	0.52	0.12
3.2.2 It is recommended to use the Rating of Perceived Exertion (RPE) scale to determine exercise intensity. This scale is simple and practical. The exercise intensity is generally set at 12–14 points (the patient feels slightly tired but can still comfortably converse without significant strain). This ensures the patient achieves safe and effective exercise outcomes without losing exercise adherence.	4.83	0.39	0.08	4.83	0.39	0.08
3.3 Exercise Duration	4.33	0.78	0.18	4.92	0.29	0.06
3.3.1 The optimal time for exercise is between 30 min and 2 h after starting dialysis.	4.42	0.52	0.12	4.08	0.29	0.07
3.3.2 The target exercise duration is 30–60 min (including warm‐up and cool‐down). At least 30 min of exercise should be completed, either in a single session or divided into multiple sessions based on the patient’s condition.	4.67	0.49	0.11	4.83	0.39	0.08
3.3.3 The exercise program should last for at least 3 months.	4.58	0.67	0.15	4.33	0.65	0.15
3.4 Exercise Type	4.58	0.52	0.11	4.33	0.65	0.15
3.4.1 A single exercise session should include warm‐up, exercise training, and cool‐down. Warm‐up: At least 5–10 min of low to moderate‐intensity aerobic and muscle endurance exercises. Exercise phase: At least 20–60 min, including aerobic exercise, resistance exercise, and flexibility exercises. Cool‐down: At least 5–10 min of low to moderate‐intensity aerobic and muscle endurance exercises.	4.33	0.49	0.11	4.08	0.52	0.13
3.4.2 During dialysis, a combination of aerobic and resistance exercises, resistance exercises alone, or aerobic exercises alone can improve dialysis adequacy. Probability ranking shows that a combination of aerobic and resistance exercises during dialysis has the best effect on improving dialysis adequacy.	4	0.43	0.11	4.50	0.52	0.12
3.4.3 It is recommended that dialysis centers use motorized bicycles with adjustable resistance for routine aerobic and resistance exercises, as this method is simple and effective. Alternatively, a combination of aerobic, resistance, and flexibility exercises in a supine position can be performed during dialysis.	4.75	0.45	0.10	4.75	0.45	0.10
3.4.4 Aerobic exercises during dialysis can include various movements of the nonarteriovenous fistula arm and both lower limbs, such as hand gripping, wrist rotation, elbow flexion and extension, and hip adduction, abduction, flexion, and extension.	4.5	0.67	0.15	4.58	0.52	0.11
3.4.5 Resistance exercises during dialysis can include lifting dumbbells with the nonfistula arm, elastic band training, progressive ankle weight training, resistance band exercises, knee extension exercises, and progressive supine leg lifts.	4.58	0.52	0.11	4.50	0.52	0.12
4 Exercise Monitoring	4.58	0.52	0.11	4.83	0.39	0.08
4.1 Monitoring Personnel	4.33	0.65	0.15	4.75	0.45	0.10
4.1.1 During the initial phase of exercise monitoring, nurses and doctors should jointly supervise. Once the patient masters the exercise method and stabilizes, nurses may monitor independently. Rehabilitation specialists should regularly participate in patient assessments and the development of subsequent exercise plans.	4.67	0.49	0.11	4.83	0.39	0.08
4.2 Monitoring Timing and Content	4.33	0.49	0.11	4.50	0.67	0.15
4.2.1 Monitoring should occur before, during, and after exercise, including vital signs and vascular access status.	4.58	0.52	0.11	4.25	0.45	0.11
4.2.2 Strengthen monitoring during dialysis and assess exercise intensity. Closely observe the patient for any discomfort during exercise and identify potential causes.	4.25	0.45	0.11	4.58	0.52	0.11
4.2.3 For diabetic patients, monitor blood glucose levels before and after exercise, as needed, to mitigate hypoglycemia risk.	4.5	0.52	0.12	4.58	0.67	0.15
4.3 Exercise Termination Criteria	4.25	0.45	0.11	4.42	0.52	0.12
4.3.1 Persistent chest or back pain, palpitations, or chest tightness.	4.5	0.52	0.12	4.50	0.52	0.12
4.3.2 Severe shortness of breath or difficulty speaking.	4.33	0.49	0.11	4.17	0.39	0.09
4.3.3 Headache, dizziness, generalized weakness, visual disturbances, or profuse sweating.	4.67	0.49	0.11	4.33	0.49	0.11
4.3.4 Severe arrhythmias.	4.5	0.52	0.12	4.58	0.52	0.11
4.3.5 Exercise‐related muscle cramps or joint pain.	4.25	0.62	0.15	4.58	0.52	0.11
5 Exercise Support	4.92	0.29	0.06	4.83	0.39	0.08
5.1 Theoretical Support	4.83	0.39	0.08	4.67	0.49	0.11
5.1.1 Use appropriate theories (e.g., Health Belief Model, Theory of Planned Behavior) to help patients adopt and adhere to exercise plans.	4.42	0.52	0.12	4.67	0.49	0.11
5.2 Social Support	4.92	0.29	0.06	4.83	0.39	0.08
5.2.1 Educate patients about exercise during dialysis, including indications, contraindications, benefits, risks, specific exercise protocols, precautions, and how to manage potential discomfort.	4.75	0.45	0.10	4.75	0.45	0.10
5.2.2 Encourage patients to use diaries, informational manuals, or other visual tools to track exercise progress. Provide demonstration materials (e.g., manuals, videos) in advance.	4.75	0.45	0.10	4.83	0.39	0.08
5.2.3 Train dialysis center staff on emergency response protocols.	4.83	0.39	0.08	4.42	0.52	0.12

#### 3.1.3. Expert Opinion Processing and Protocol Modification Process

The criteria for retaining indicators were defined as follows: only items with mean scores for both importance and operability ≥ 3.5 and a CV < 0.25 were retained [41]. After completing the first round of consultation, the research team revised the intervention protocol in accordance with these screening criteria and the experts’ recommendations. The specific revisions are summarized below.

##### 3.1.3.1. Addition of Items

• Under the “Exercise Monitoring” dimension, a new secondary item entitled “Monitoring Personnel” was added, along with corresponding tertiary items specifying the responsibilities of the monitoring staff. This revision was made in response to recommendations from four experts who emphasized the need to clearly define responsibility for monitoring exercise safety.
• Under “Patient Preparation” within the “Pre‐exercise Preparation” dimension, the following tertiary item was added: “Ensure adequate dialysis”. Clinical conditions such as volume overload, metabolic acidosis, anemia, malnutrition, and electrolyte imbalance may increase the risks associated with exercise implementation.” This addition reflected the opinions of three experts, who highlighted the importance of controlling baseline clinical risks and noted that maintaining adequate dialysis duration could help reduce them.
• Under “Monitoring Content and Timing” in the “Exercise Monitoring” dimension, the following tertiary item was added: “For patients with diabetes, attention should be given to the risk of hypoglycemia, and blood glucose levels should be monitored before and after exercise when necessary.” This modification incorporated suggestions from two experts regarding enhanced safety monitoring for special populations, particularly patients with diabetes.

##### 3.1.3.2. Modification of Items

• The item “Exercise capacity assessment: Determination of maximal oxygen uptake (maximal oxygen)” was revised to: “Exercise capacity assessment: As more than 50% of MHD patients have difficulty completing maximal oxygen uptake testing, the use of simple physical function assessments, such as the 6 min walk test, is recommended.” This revision was made because the original item did not meet the indicator screening criteria and because three experts emphasized the importance of using assessment methods that are practical and feasible for the target population.
• The item “Exercise frequency: 3 times per week” was revised to “Exercise frequency should be determined according to the patient’s dialysis schedule, with a recommended frequency of 2–3 times per week.” This modification was based on the item’s failure to meet the screening criteria and incorporated expert recommendations to better align the exercise frequency with dialysis treatment schedules.

These revisions were incorporated into the questionnaire for the second round of expert consultation. After the second round, expert opinions showed a clear trend toward convergence, as reflected in the further reduction in the coefficients of variation across all indicators and the absence of any major new disagreements. Therefore, the consultation process was concluded. Based on additional suggestions provided during the second round, the research team further discussed and added a tertiary item under the secondary item “Social Support” within the “Exercise Support” dimension: “Strengthen education and awareness among patients’ family members to encourage them to provide greater support for the patients.” This addition was intended to enhance the supportive role of family involvement in exercise interventions. The finalized intervention program consists of 5 primary items, 14 secondary items, and 44 tertiary items, as presented in Table 8. Furthermore, to facilitate standardized, sustainable implementation of intradialytic exercise and to translate the intervention program into practical daily nursing activities, this study developed a set of safe, reproducible, and progressively adjustable clinical operational procedures based on the exercise protocol. These procedures provide a foundation for future research and offer a reference for healthcare professionals. Detailed information is provided in Appendix 3.

**TABLE 8:** The final exercise intervention plan for maintenance hemodialysis patients.

Primary indicator	Second indicator	Tertiary indicator	Level of evidence	Quality of evidence	HBM domains
1. Necessity of Exercise	1.1. Exercise Safety	1.1.1. Exercise during dialysis is safe, and it is recommended that all treatment institutions provide exercise during dialysis to assist in treatment.	++	A	Perceived Benefits
	1.2. Exercise Benefits	1.2.1. Exercising for at least 60 min three times a week during dialysis can improve the survival rate of dialysis patients.	++++	B	
		1.2.2. Moderate‐intensity resistance and aerobic exercise during dialysis can improve dialysis adequacy.	++++	A	
		1.2.3. Exercise during dialysis can significantly improve microinflammatory status, serum albumin levels, fatigue, and sleep conditions in dialysis patients.	++++	A	
		1.2.4. Exercise during dialysis can improve physical function, quality of life, cardiopulmonary function, and blood pressure levels in dialysis patients.	+++	B	
		1.2.5. Exercise during dialysis can alleviate psychological disorders such as depression and anxiety in patients.	+++	B	
		1.2.6. It is worth emphasizing that even a slight increase in physical activity levels may be beneficial.	++++	A	

2. Pre‐Exercise Preparation	2.1. Preparations for Research Personnel	2.1.1. It is recommended that professional medical staff develop the exercise plan and implement it accordingly.	+++	A	Perceived Barriers
		2.1.2. Researchers should enhance their knowledge of relevant expertise, educate dialysis patients and their families about the implementation methods, safety monitoring, and outcome evaluation, and fully recognize its clinical benefits.	++++	A	
	2.2. Preparations for Patients	2.2.1. Patients should be informed of the benefits and risks before exercise, and informed consent should be obtained from the patient/family, with a signed consent form.	+++	A	
		2.2.2. Ensure the patency of vascular access, as it is the lifeline for dialysis patients and a necessary condition for adequate dialysis.	++++	A	
		2.2.3. Ensure adequate dialysis. Clinical issues such as volume overload, metabolic acidosis, anemia, malnutrition, and electrolyte imbalances often increase the risks of exercise implementation.	+++	A	
	2.3. Exercise Safety Assessment	2.3.1. Clinical Condition Assessment: ① Medical history assessment: Symptoms, comorbidities (especially cardiovascular diseases, bone and joint abnormalities, etc.), medication history, lifestyle habits, exercise habits, family history, etc. ② Physical examination ③ Laboratory tests.	++++	B	
		2.3.2. Exercise Capacity Assessment: Since over 50% of MHD patients cannot complete maximal oxygen uptake (VO2max) testing, it is recommended to use simple physical function tests, such as the 6‐min walk test.	+++	A	
	2.4. Exercise Contraindications	2.4.1. Abnormal Blood Pressure: Severe hypertension (blood pressure exceeding 180/110 mmHg) or hypotension (blood pressure below 90/60 mmHg).	++++	A	
		2.4.2. Cardiopulmonary Diseases: Severe heart failure, arrhythmias, unstable angina, severe pericardial effusion, valvular stenosis, hypertrophic cardiomyopathy, aortic dissection, uncontrolled pulmonary hypertension (mean pulmonary artery pressure > 55 mmHg).	++++	A	
		2.4.3. Acute Clinical Events: Acute systemic inflammatory diseases or fever, acute phase of cardiovascular or cerebrovascular diseases, acute phase of traumatic injuries, etc.	++++	A	
		2.4.4. New‐Onset Deep Vein Thrombosis Symptoms: Exercise should be postponed or stopped if symptoms such as abnormal redness, swelling, or pain in the calf occur.	++++	A	
		2.4.5. Patient Inability to Cooperate with Exercise: If the patient is unable to cooperate with the exercise program.	++++	A	

3. Exercise Prescription	3.1. Exercise Frequency	3.1.1. Exercise frequency should be 2–3 times per week, based on the patient’s dialysis schedule.	++	A	Action
	3.2. Exercise Intensity	3.2.1. Start with low‐intensity exercise and gradually progress to moderate‐intensity exercise.	+++	B	
		3.2.2. It is recommended to use the Rating of Perceived Exertion (RPE) scale to determine exercise intensity. This scale is simple and practical. The exercise intensity is generally set at 12–14 points (the patient feels slightly tired but can still comfortably converse without significant strain). This ensures the patient achieves safe and effective exercise outcomes without losing exercise adherence.	++++	A	
	3.3. Exercise Duration	3.3.1. The optimal time for exercise is between 30 min and 2 h after starting dialysis.	++++	A	
		3.3.2. The target exercise duration is 30–60 min (including warm‐up and cool‐down). At least 30 min of exercise should be completed, either in a single session or divided into multiple sessions, depending on the patient’s condition.	+++	A	
		3.3.3. The exercise program should last for at least 3 months.	++	B	
	3.4. Type of Exercise	3.4.1. A single exercise session should include warm‐up, exercise training, and cool‐down. Warm‐up: At least 5–10 min of low to moderate‐intensity aerobic and muscle endurance exercises. Exercise phase: At least 20–60 min, including aerobic exercise, resistance exercise, and flexibility exercises. Cool‐down: At least 5–10 min of low to moderate‐intensity aerobic and muscle endurance exercises.	++++	A	
		3.4.2. During dialysis, a combination of aerobic and resistance exercises, resistance exercises alone, or aerobic exercises alone can improve dialysis adequacy. Probability ranking shows that a combination of aerobic and resistance exercises during dialysis has the best effect on improving dialysis adequacy.	++++	A	
		3.4.3. It is recommended that dialysis centers use motorized bicycles with adjustable resistance for routine aerobic and resistance exercises, as this method is simple and effective. Alternatively, a combination of aerobic, resistance, and flexibility exercises in a supine position can be performed during dialysis.	+++	B	
		3.4.4. Aerobic exercises during dialysis can include various movements of the nonarteriovenous fistula arm and both lower limbs, such as hand gripping, wrist rotation, elbow flexion and extension, and hip adduction, abduction, flexion, and extension.	++++	A	
		3.4.5, Resistance exercises during dialysis can include lifting dumbbells with the nonfistula arm, elastic band training, progressive ankle weight training, resistance band exercises, knee extension exercises, and progressive supine leg lifts.	+++	B	

4. Exercise Monitoring	4.1. Monitoring Personnel	4.1.1. During the initial phase of exercise monitoring, nurses and doctors should jointly supervise. Once the patient masters the exercise method and stabilizes, nurses may monitor independently. Rehabilitation specialists should regularly participate in patient assessments and the development of subsequent exercise plans.	+++	B	Perceived Barriers
	4.2. Timing and Content of Monitoring	4.2.1. Monitoring should occur before, during, and after exercise, including vital signs and vascular access status.	++++	A	
		4.2.2. Strengthen monitoring during dialysis and assess exercise intensity. Closely observe the patient for any discomfort during exercise and identify potential causes.	++++	A	
		4.2.3, For diabetic patients, monitor blood glucose levels before and after exercise, as needed, to mitigate hypoglycemia risk.	++	B	
	4.3. Indications for Exercise Termination	4.3.1. Persistent chest or back pain, palpitations, or chest tightness.	++++	A	
		4.3.2. Severe shortness of breath or difficulty speaking.	++++	A	
		4.3.3. Headache, dizziness, generalized weakness, visual disturbances, or profuse sweating.	++++	A	
		4.3.4. Severe arrhythmias.	++++	A	
		4.3.5. Exercise‐related muscle cramps or joint pain.	++++	A	

5. Exercise Support	5.1. Theoretical Support	5.1.1. Use appropriate theories (e.g., Health Belief Model, Theory of Planned Behavior) to help patients adopt and adhere to exercise plans.	++	A	Cues to Action
	5.2. Social Support	5.2.1. Educate patients about exercise during dialysis, including indications, contraindications, benefits, risks, specific exercise protocols, precautions, and how to manage potential discomfort.	+++	A	
		5.2.2. Encourage patients to use diaries, informational manuals, or other visual tools to track exercise progress. Provide demonstration materials (e.g., manuals, videos) in advance.	++++	A	
		5.2. 3. Train dialysis center staff on emergency response protocols.	+++	A	
		5.2.4. Strengthen education and awareness efforts for patients’ family members to enable them to provide greater support for the patients.	+++	B	

## 4. Discussion

### 4.1. Demand for Intradialytic Exercise Interventions in Maintenance Hemodialysis Patients

Patients undergoing MHD frequently experience complications such as microinflammatory states, muscle atrophy, cardiovascular disorders, and frailty as a consequence of renal failure and prolonged dialysis treatment. These pathological changes significantly impair patients’ quality of life and negatively affect clinical outcomes and prognosis [42–44]. For example, the prevalence of microinflammatory states among MHD patients has been reported to range from 30% to 50%, typically characterized by persistently elevated inflammatory markers, including CRP, IL‐6, and TNF‐α. Previous studies have demonstrated that regular exercise can alleviate microinflammatory responses by reducing oxidative stress products and enhancing antioxidant capacity [45, 46]. Additionally, frailty is highly prevalent among MHD patients, with reported rates ranging from 14% to 73%. Exercise interventions have been shown to reduce frailty severity while improving muscle strength and physical mobility [47]. Intradialytic exercise may also enhance solute clearance, improve dialysis adequacy, and help correct electrolyte imbalances, including reductions in serum phosphorus levels [48, 49]. These findings indicate that exercise interventions, as a nonpharmacological therapeutic strategy, can effectively alleviate multiple complications associated with MHD. Despite the recognized benefits of exercise, current exercise participation among MHD patients remains inadequate, and many challenges persist in the clinical implementation of exercise interventions for this population. Survey data indicate that approximately 69% of patients engage in exercise only occasionally, while the proportion of patients performing regular exercise is as low as 47.2%. Moreover, nearly 76.8% of patients possess limited knowledge regarding exercise [50]. Several factors influence exercise participation, including age, comorbid conditions, physical functional status, concerns regarding exercise safety, insufficient professional guidance, fatigue, perceived benefits and barriers, and levels of exercise self‐efficacy. Furthermore, substantial individual differences exist in patients’ preferences regarding exercise type, intensity, and frequency, highlighting the need to consider factors such as age, complications, and individual tolerance when designing exercise programs. Therefore, this study aimed to develop an intradialytic exercise intervention program for MHD patients based on the HBM. Through individualized assessment and tailored exercise interventions, the program seeks to improve patients’ awareness of exercise, strengthen self‐management abilities, and enhance exercise adherence and compliance.

### 4.2. The Innovativeness of Constructing an Exercise Program During Dialysis Based on the HBM

Previous studies have shown that the overall exercise participation of MHD patients remains inadequate, with generally poor exercise adherence [24]. Moreover, the worldwide implementation rate of intradialytic exercise is reported to be below 10% [51]. Factors influencing exercise behavior in MHD patients include physiological, psychological, and socio‐cultural dimensions. Compared with physiological limitations, psychological factors are considered more modifiable and therefore may provide a more practical target for intervention strategies [52]. Current evidence suggests that exercise participation among MHD patients is mainly influenced by four psychological factors: perceived benefits of exercise, perceived barriers to exercise, exercise self‐efficacy, and social support. In particular, patients’ perceptions of the value of exercise and the barriers to it play a critical role in exercise‐related decision‐making [53]. MHD patients who regularly engage in exercise tend to demonstrate greater exercise confidence and stronger self‐efficacy, both of which are positively associated with exercise adherence and are considered important predictors of long‐term exercise maintenance [6]. Stronger social support networks have been positively linked to exercise participation. Adequate support from family members, healthcare providers, and peers can enhance patients’ motivation through positive reinforcement and encourage the adoption of healthier behaviors. These findings indicate that interventions aimed at strengthening self‐efficacy and improving social support systems may effectively enhance exercise adherence among MHD patients. The HBM explains health behavior change from a psychosocial perspective. Accordingly, this study developed an intradialytic exercise intervention program for MHD patients based on the HBM framework. During program development, full consideration was given to the major factors influencing exercise‐related health behaviors in MHD patients, including limited awareness of exercise benefits, perceived barriers to exercise, insufficient knowledge regarding healthy behaviors, low self‐efficacy, and inadequate behavioral cues or guidance. Guided by these considerations, the final intervention framework was structured into five major components: the necessity of exercise, pre‐exercise preparation, exercise prescription, exercise monitoring, and exercise support. Therefore, the HBM not only served as the theoretical basis for the intervention program but also directly informed its structural design, with the ultimate goal of promoting the initiation and long‐term maintenance of healthy exercise behaviors among MHD patients.

### 4.3. The Comprehensiveness of Constructing an Exercise Program During Dialysis Based on the HBM

To enhance the clinical applicability of the program, this study comprehensively considered all major aspects of implementing exercise therapy. Guided by the HBM, five core components were established: the necessity of exercise, pre‐exercise preparation, exercise prescription, exercise monitoring, and exercise support. The “necessity of exercise” component mainly focuses on two aspects: safety and therapeutic benefits. While encouraging MHD patients to participate in exercise, potential risks must also be carefully addressed. Therefore, ensuring the safety and effectiveness of exercise interventions is essential, and comprehensive safety assessments for MHD patients should be conducted before exercise implementation. The “pre‐exercise preparation” component includes preparation by both healthcare professionals and patients, as well as safety evaluations. Previous studies have shown [54] that insufficient knowledge among nurses represents a major barrier to the implementation of intradialytic exercise for MHD patients. Therefore, the development of this program emphasized not only patient preparation before exercise but also professional training for healthcare providers regarding exercise during dialysis. This approach aims to enhance nurses’ knowledge and initiative, establishing a strong foundation for the effective implementation of exercise therapy. The “exercise prescription” component was structured into four secondary categories according to the FITT principle, providing detailed parameters to guide exercise implementation. Further, exercise prescriptions should be individualized based on patient tolerance, dialysis center safety conditions, and the home environment. A combination of different exercise modalities is recommended to maximize overall therapeutic benefits. The “exercise monitoring” component includes three secondary aspects: monitoring personnel, monitoring timing and content, and indications for exercise termination. To ensure the overall safety of exercise therapy, the program recommends that exercise during dialysis be supervised by professionally trained healthcare personnel through multidisciplinary collaboration, maximizing therapeutic effectiveness while minimizing potential risks. The “exercise support” component consists of two secondary indicators: theoretical support and social support. Previous research has demonstrated that social support is positively associated with exercise behavior [55]. Higher levels of social support provide patients with greater encouragement and assistance, strengthening their motivation to engage in positive health behaviors. Therefore, the long‐term maintenance of exercise therapy requires not only adequate theoretical guidance but also sustained support from healthcare institutions and family members. The program was developed from multiple perspectives, including health education, safety monitoring, and support systems, ensuring its comprehensiveness and facilitating the establishment and maintenance of long‐term exercise habits among MHD patients.

### 4.4. The Scientific Nature of Constructing an Exercise Program During Dialysis Based on the HBM

This study was conducted based on a comprehensive literature review and strictly adhered to the principles and procedures of evidence‐based practice, ensuring the rigor of the program development process. During literature retrieval, the evidence pyramid model was followed, with priority given to high‐quality evidence sources, resulting in a high overall methodological quality of the included studies. Most of the included literature was published recently, further strengthening the reliability and relevance of the evidence. To ensure that the evidence synthesis was scientifically rigorous, clinically applicable, and practically operable within current healthcare settings, this study also incorporated expert consultation. Opinions and recommendations from experts in relevant fields were collected and integrated into the program development process. Through two rounds of Delphi expert consultation, the preliminary intradialytic exercise program for MHD patients was continuously refined and optimized, ultimately enhancing the scientific validity of the program and providing a more reliable basis for future clinical practice.

### 4.5. The Feasibility of Constructing an Exercise Program During Dialysis Based on the HBM

The long‐term implementation of an intervention protocol depends on successfully translating “knowing what to do” into “being able to implement it consistently in practice.” Implementing this protocol requires coordinated management of several key factors. First, human resources and training requirements must be considered. The protocol recommends that exercise prescription and monitoring be conducted by professionally trained healthcare personnel. This requires systematic training for dialysis nurses and physicians to develop competencies in exercise prescription, exercise intensity assessment, and emergency management during exercise interventions. Therefore, both the initial workforce investment and the ongoing costs associated with professional training should be considered by healthcare institutions. Second, considerations related to equipment and spatial organization are also important. The protocol recommends exercise modalities such as motorized stationary cycling and supine exercise routines, which involve issues related to equipment procurement, maintenance, and appropriate arrangement within the dialysis unit. For dialysis centers with limited space, priority may be given to supine exercise programs that do not require large equipment. However, it remains essential to ensure that these exercise approaches are both effective and engaging to maintain patient adherence and participation. Finally, integrating exercise interventions into the existing dialysis workflow presents an important operational challenge, particularly in busy clinical settings. Effective implementation requires clear coordination between scheduled exercise periods, such as 30 min to 2 h after dialysis initiation, as specified in the protocol, and routine nursing activities, including blood pressure monitoring and patient rounds. Such coordination is necessary to ensure the quality, safety, and continuity of exercise monitoring throughout the dialysis session.

### 4.6. The Safety of Constructing an Exercise Program During Dialysis Based on the HBM

Previous studies [56, 57] have demonstrated that exercise can simultaneously improve both physical and psychological health. Compared with most other nonpharmacological interventions, exercise addresses a wider range of symptoms commonly experienced by hemodialysis patients, making it a highly effective and comprehensive strategy for improving overall patient well‐being. However, the ultimate value of any clinical intervention program depends on its safety and feasibility in real‐world clinical settings. In the present protocol, detailed recommendations regarding exercise timing, monitoring content, and exercise termination criteria were established to provide a clear operational framework for ensuring patient safety, with particular emphasis on minimizing exercise‐related risks such as serious cardiovascular events. However, this study currently lacks real‐world safety data derived from the practical implementation of the protocol. Therefore, a key objective of future clinical practice and research will be the systematic collection and reporting of adverse event incidence associated with intradialytic exercise interventions. Future studies will focus on establishing standardized safety monitoring procedures, documenting all adverse events, and conducting comparative analyses with nonexercise control groups. The proposed safety monitoring framework includes the following components: (1) Types of monitored events: including, but not limited to, hypotension, hypertension, arrhythmias, muscle cramps, falls, and vascular access injury; (2) monitoring frequency: real‐time monitoring during each exercise session conducted during dialysis, with vital signs and subjective symptoms recorded before exercise, during exercise (every 30 min), and after exercise; and (3) reporting procedures: all adverse events will be graded and documented according to internationally recognized standards, such as the Common Terminology Criteria for Adverse Events (CTCAE), and periodically reviewed by an independent Data Safety Monitoring Board. These measures will provide stronger evidence supporting the safety and controllability of protocol implementation under professional supervision, increasing confidence among both healthcare providers and patients regarding the use of intradialytic exercise interventions.

### 4.7. Implications for Nursing Managers

This study demonstrates that an intradialytic exercise intervention based on the HBM can effectively improve exercise adherence and self‐efficacy among MHD patients. From a nursing management perspective, several practical considerations warrant attention. First, managers should integrate the HBM into staff training programs to enhance nurses’ understanding of the value of exercise interventions and improve their patient communication skills. Second, a standardized, unit‐level protocol for intradialytic exercise should be developed—including pre‐exercise assessment, intraexercise monitoring, and postexercise documentation—with clearly assigned responsibilities. Third, a continuous quality improvement mechanism should be established, for example, by tracking monthly exercise participation rates, adverse events, and patient feedback to refine the intervention accordingly. Fourth, support from hospital administration should be sought to secure resources such as simple exercise equipment (e.g., pedal exercisers and resistance bands) and to incorporate exercise‐related metrics into the nursing quality evaluation system. These management strategies can help overcome the prevailing “treatment over rehabilitation” mindset in many hemodialysis units and facilitate the translation of intradialytic exercise interventions from research into routine practice.

## 5. Limitations

This study has several limitations. First, due to funding and time constraints, the Delphi consultation process involved only experts from China. Although the findings may still provide valuable reference for other countries and regions, broader international expert participation could further enhance the program’s generalizability. Second, during the development of the exercise intervention program, literature retrieval and selection were conducted according to the evidence pyramid model, with the final evidence base consisting primarily of high‐level evidence such as guidelines and systematic reviews. Although this strengthened the program’s scientific rigor, it also yielded an intervention framework that remains relatively general and lacks detailed operational guidance in some areas. Future studies should incorporate evidence from randomized controlled trials and qualitative research to further refine the intervention program, particularly regarding practical implementation details. Finally, this study focused primarily on the theoretical development of an exercise intervention program for MHD patients. The clinical effectiveness, feasibility, and applicability of the program have not yet been validated in practice. Therefore, future clinical studies are needed to evaluate the program in real‐world settings and to continuously optimize and improve the intervention based on practical implementation outcomes.

## 6. Conclusion

This study comprehensively considered the current exercise status of MHD patients in China and the various factors influencing exercise participation. Based on the HBM, an intradialytic exercise intervention program for MHD patients was developed with consideration of the entire exercise implementation process, demonstrating both innovation and comprehensiveness. The development of the intervention protocol was guided by evidence‐based medicine principles, ensuring its scientific rigor and providing an important reference for the implementation and clinical guidance of exercise interventions during dialysis in MHD patients. During practical application, healthcare professionals should comprehensively evaluate patients’ psychological, physiological, and social conditions while also considering the clinical setting and environmental factors related to exercise implementation. Exercise interventions should therefore be individualized according to available evidence, patient characteristics, and local clinical conditions. Finally, because the present intervention program was developed within a specific theoretical framework, further studies are needed to determine whether the protocol can effectively improve patient adherence and long‐term compliance with exercise interventions. Future research will provide more robust clinical evidence to support the broader application of this program in clinical practice.

## Author Contributions

Xianjuan Cheng: conceptualization, data curation, formal analysis, investigation, methodology, visualization, and writing–original draft Preparation. Heng Dai: investigation, project administration, resources, and supervision. Yuanchun Guan: investigation, resources, and supervision. Chunling Xia: resources and supervision. Hailong Hou: investigation, data curation, and validation. He Li: investigation, data curation, and validation. Hangfei Qu: writing–review and editing. Shiqi Xiao: conceptualization, investigation, methodology, project administration, validation, supervision, and writing–review and editing.

## Funding

The authors have nothing to report.

## Ethics Statement

This manuscript is an analytical study based on existing literature and expert opinions. It does not involve original human/animal experimentation, personal privacy, or sensitive data; therefore, ethics committee approval was not required. This study has obtained informed consent from the Delphi study experts. As further empirical research is required subsequently, the study has received approval from the Ethics Committee and has been registered with the Chinese Clinical Trial Registry. The registration number is ChiCTR2500113004. All referenced content has been properly cited in accordance with academic standards and complies with copyright and fair use principles.

## Conflicts of Interest

The authors declare no conflicts of interest.

## Supporting Information

Additional supporting information can be found online in the Supporting Information section.

## Supporting information


**Supporting Information** Appendix 1: Development of an Exercise Intervention Program During Dialysis for Patients on Maintenance Hemodialysis Based on the Health Belief Model. Expert Consultation Questionnaire (Round 1). Appendix 2: Development of an Exercise Intervention Program During Dialysis for Patients on Maintenance Hemodialysis Based on the Health Belief Model. Expert Consultation Questionnaire (Round 2). Appendix 3: Specific Operational Procedures for Exercise Intervention During Dialysis Based on the Health Belief Model.

## Data Availability

The data that support the findings of this study are available from the corresponding authors upon reasonable request.
