# Supplementary material for: Development of an Exercise Intervention Program During Dialysis for Patients on Maintenance Hemodialysis Based on the Health Belief Model
Source: J Nurs Manag. 2026 Jul 10;2026:5514298. doi: 10.1155/jonm/5514298 (PMC13351614; doi:10.1155/jonm/5514298)
Supplement: Supplementary file 1 — Supporting Information Appendix 1: Development of an Exercise Intervention Program During Dialysis for Patients on Maintenance Hemodialysis Based on the Health Belief Model. Expert Consultation Questionnaire (Round 1). Appendix 2: Development of an Exercise Intervention Program During Dialysis for Patients on Maintenance Hemodialysis Based on the Health Belief Model. Expert Consultation Questionnaire (Round 2). Appendix 3: Specific Operational Procedures for Exercise Intervention During Dialysis Based on the Health Belief Model. [file JONM-2026-5514298-s001.doc]

**Appendix 1：**

**Development of an Exercise Intervention Program During Dialysis for Patients on Maintenance Hemodialysis Based on the Health Belief Model**

**Expert Consultation Questionnaire (Round 1)**

Dear Experts,

It is my great honor to invite you to serve as a consulting expert for the development of an exercise intervention program for patients on maintenance hemododialysis. My name is Cheng Xianjuan, from Shengjing Hospital of China Medical University,. My research topic is "Development of an Exercise Intervention Program During Dialysis for Patients on Maintenance Hemodialysis Based on the Health Belief Model". Given your distinguished expertise in this field, I cordially invite you to participate as a consulting expert for this study. Below is a brief introduction to the research background:

In recent years, numerous domestic and international studies have demonstrated that exercise training during dialysis can improve the adequacy of dialysis, mitigate micro-inflammatory states, enhance physical function, alleviate psychological conditions, and improve the quality of daily life for patients on maintenance hemododialysis (MHD). However, most existing research on exercise interventions during dialysis for MHD patients focuses on the effects of single factors on related outcomes. Moreover, there is limited evidence-based summary or guideline development for exercise management protocols; only in the past two years have relevant evidence summaries begun to emerge. Overall, exercise engagement among MHD patients remains suboptimal. To address this issue, some scholars have suggested applying appropriate theoretical models, such as the Health Belief Model or the Theory of Planned Behavior, to facilitate the adoption and adherence of exercise programs. Therefore, this study aims to develop an evidence-based exercise intervention program for MHD patients grounded in the Health Belief Model. The program seeks to provide a foundation for implementing exercise interventions for MHD patients, enhance their exercise adherence, and ultimately improve dialysis adequacy, reduce anxiety and depression, and elevate their quality of life. Based on preliminary groundwork and literature review, we have formulated a draft exercise intervention program for MHD patients, consisting of 5 primary domains (the necessity of exercise, exercise safety assessment, exercise prescription, exercise monitoring, and exercise support), 14 secondary items, and 40 tertiary items.

This expert consultation questionnaire includes three sections: (1) Expert Background Information Survey, (2) Expert Consultation Questionnaire on the Exercise Intervention Program for MHD Patients, and (3) Self-assessment Questionnaire on the Expert's Familiarity (Cs) and Basis of Judgment (Ca) regarding the program. Please fill out the forms and provide your feedback truthfully after reading the corresponding instructions. Additionally, all information you provide will be used solely for research purposes. The research results will be presented in academic publications, and your personal details will not be disclosed in any form during the writing or publication process.

We sincerely value your insightful suggestions. Your support is crucial to the successful progression of this research project. Thank you for taking the time from your busy schedule. Wishing you a productive and pleasant work experience!

Sincerely,

Cheng Xianjuan

Affiliation: Shengjing Hospital, China Medical University

Phone: 18940252396

Email: [1127200419@qq.com](mailto:1127200419@qq.com)

**Part I Expert Background Information Survey**

Instructions:To ensure the rigor of this study,please complete the following items based on your actual situation. Place a check mark (√) in the appropriate option. Thank you sincerely for your support of this research project!

| 1. Age：____ |
| --- |
| 1. Gender：Male Female |
| 1. Highest Degree Obtained：Bachelor's□ Master's or above |
| 1. Academic/Professional Title：Junior□ Intermediate□ Senior or above□ |
| 1. Years of Professional Experience: _____ years |
| 1. Research Field(s)：Nephrology□ Clinical Nursing□ Exercise Rehabilitation |

**Part II Expert Consultation Questionnaire on the Exercise Intervention Program**

Instructions for Completion:Please evaluate theimportance and operational feasibility of each item using the Likert 5-point scale. Scores range from 1 to 5 points, where 1 point indicates "Not Important/Not Feasible" and 5 points indicates "Very Important/Very Feasible".If you find the description of any indicator inaccurate or believe it should be deleted,please specify your reasons in the "Specific Revision Suggestions" column.If you believe there are relevant elements not yet considered in this study that should be added,please provide your additions in the "Additional Items" column.

Table 1 Consultation Form for Primary Indicators

| Primary Indicator | Item Importance | | | | | Item Operational Feasibility | | | | | Specific Revision Suggestions |
| --- | --- | --- | --- | --- | --- | --- | --- | --- | --- | --- | --- |
|  | 5  Very Important | 4  Fairly Important | 3  Moderately Important | 2  Slightly Important | 1  Not Important | 5  Very Strong | 4  Fairly Strong | 3  Moderate | 2  Slightly Strong | 1  Not Strong |  |
| 1.Necessity of Exercise |  |  |  |  |  |  |  |  |  |  |  |
| 2.Pre-Exercise Preparation |  |  |  |  |  |  |  |  |  |  |  |
| 3.Exercise Prescription |  |  |  |  |  |  |  |  |  |  |  |
| 4.Exercise Monitoring |  |  |  |  |  |  |  |  |  |  |  |
| 5.Exercise Support |  |  |  |  |  |  |  |  |  |  |  |

For Table 1, if you have any other comments or suggestions, please note them here: __________________________________________________________________________________

Table 2 Consultation Form for Second Indicators

| Second Indicator | Item Importance | | | | | Item Operational Feasibility | | | | | Specific Revision Suggestions |
| --- | --- | --- | --- | --- | --- | --- | --- | --- | --- | --- | --- |
|  | 5  Very Important | 4  Fairly Important | 3  Moderately Important | 2  Slightly Important | 1  Not Important | 5  Very Strong | 4  Fairly Strong | 3  Moderate | 2  Slightly Strong | 1  Not Strong |  |
| 1.1 Exercise Safety |  |  |  |  |  |  |  |  |  |  |  |
| 1.2 Exercise Benefits |  |  |  |  |  |  |  |  |  |  |  |
| 2.1 Preparations for Research Personnel |  |  |  |  |  |  |  |  |  |  |  |
| 2.2 Preparations for Patients |  |  |  |  |  |  |  |  |  |  |  |
| 2.3 Exercise Safety Assessment |  |  |  |  |  |  |  |  |  |  |  |
| 2.4 Exercise Contraindications |  |  |  |  |  |  |  |  |  |  |  |
| 3.1 Exercise Frequency |  |  |  |  |  |  |  |  |  |  |  |
| 3.2 Exercise Intensity |  |  |  |  |  |  |  |  |  |  |  |
| 3.3 Exercise Duration |  |  |  |  |  |  |  |  |  |  |  |
| 3.4 Type of Exercise |  |  |  |  |  |  |  |  |  |  |  |
| 4.1 Timing and Content of Monitoring |  |  |  |  |  |  |  |  |  |  |  |
| 4.2 Indications for Exercise Termination |  |  |  |  |  |  |  |  |  |  |  |
| 5.1 Theoretical Support |  |  |  |  |  |  |  |  |  |  |  |
| 5.2 Social Support |  |  |  |  |  |  |  |  |  |  |  |

For Table 2, if you have any other comments or suggestions, please note them here: ___________________________________________________________________

Table 3 Consultation Form for Tertiary Indicators

| Tertiary Indicator | Item Importance | | | | | Item Operational Feasibility | | | | | Specific Revision Suggestions |
| --- | --- | --- | --- | --- | --- | --- | --- | --- | --- | --- | --- |
|  | 5  Very Important | 4  Fairly Important | 3  Moderately Important | 2  Slightly Important | 1  Not Important | 5  Very Strong | 4  Fairly Strong | 3  Moderate | 2  Slightly Strong | 1  Not Strong |  |
| 1.1.1 Exercise during dialysis is safe, and it is recommended that all treatment institutions provide exercise during dialysis to assist in treatment. |  |  |  |  |  |  |  |  |  |  |  |
| 1.2.1 Exercising for at least 60 minutes three times a week during dialysis can improve the survival rate of dialysis patients. |  |  |  |  |  |  |  |  |  |  |  |
| 1.2.2 Moderate-intensity resistance and aerobic exercise during dialysis can improve dialysis adequacy. |  |  |  |  |  |  |  |  |  |  |  |
| 1.2.3 Exercise during dialysis can significantly improve micro-inflammatory status, serum albumin levels, fatigue, and sleep conditions in dialysis patients. |  |  |  |  |  |  |  |  |  |  |  |
| 1.2.4 Exercise during dialysis can improve physical function, quality of life, cardiopulmonary function, and blood pressure levels in dialysis patients. |  |  |  |  |  |  |  |  |  |  |  |
| 1.2.5 Exercise during dialysis can alleviate psychological disorders such as depression and anxiety in patients. |  |  |  |  |  |  |  |  |  |  |  |
| 1.2.6 It is worth emphasizing that even a slight increase in physical activity levels may be beneficial. |  |  |  |  |  |  |  |  |  |  |  |
| 2.1.1 It is recommended that professional medical staff develop the exercise plan and implement it accordingly. |  |  |  |  |  |  |  |  |  |  |  |
| 2.1.2 Researchers should enhance their knowledge of relevant expertise, educate dialysis patients and their families about the implementation methods, safety monitoring, and outcome evaluation, and fully recognize its clinical benefits. |  |  |  |  |  |  |  |  |  |  |  |
| 2.2.1 Patients should be informed of the benefits and risks before exercise, and informed consent should be obtained from the patient/family, with a signed consent form. |  |  |  |  |  |  |  |  |  |  |  |
| 2.2.2 Ensure the patency of vascular access, as it is the lifeline for dialysis patients and a necessary condition for adequate dialysis. |  |  |  |  |  |  |  |  |  |  |  |
| 2.3.1 Clinical Condition Assessment: ① Medical history assessment: Symptoms, comorbidities (especially cardiovascular diseases, bone and joint abnormalities, etc.), medication history, lifestyle habits, exercise habits, family history, etc. ② Physical examination ③ Laboratory tests. |  |  |  |  |  |  |  |  |  |  |  |
| 2.3.2 Exercise Capacity Assessment: Measurement of Maximal Oxygen Uptake (VO₂max). |  |  |  |  |  |  |  |  |  |  |  |
| 2.4.1 Abnormal Blood Pressure: Severe hypertension (blood pressure exceeding 180/110 mmHg) or hypotension (blood pressure below 90/60 mmHg). |  |  |  |  |  |  |  |  |  |  |  |
| 2.4.2 Cardiopulmonary Diseases: Severe heart failure, arrhythmias, unstable angina, severe pericardial effusion, valvular stenosis, hypertrophic cardiomyopathy, aortic dissection, uncontrolled pulmonary hypertension (mean pulmonary artery pressure > 55 mmHg). |  |  |  |  |  |  |  |  |  |  |  |
| 2.4.3 Acute Clinical Events: Acute systemic inflammatory diseases or fever, acute phase of cardiovascular or cerebrovascular diseases, acute phase of traumatic injuries, etc. |  |  |  |  |  |  |  |  |  |  |  |
| 2.4.4 New-Onset Deep Vein Thrombosis Symptoms: Exercise should be postponed or stopped if symptoms such as abnormal redness, swelling, or pain in the calf occur. |  |  |  |  |  |  |  |  |  |  |  |
| 2.4.5 Patient Inability to Cooperate with Exercise: If the patient is unable to cooperate with the exercise program. |  |  |  |  |  |  |  |  |  |  |  |
| 3.1.1 Exercise frequency should be 3 times per week. |  |  |  |  |  |  |  |  |  |  |  |
| 3.2.1 Start with low-intensity exercise and gradually progress to moderate-intensity exercise. |  |  |  |  |  |  |  |  |  |  |  |
| 3.2.2 It is recommended to use the Rating of Perceived Exertion (RPE) scale to determine exercise intensity. This scale is simple and practical. The exercise intensity is generally set at 12～14 points (the patient feels slightly tired but can still comfortably converse without significant strain). This ensures the patient achieves safe and effective exercise outcomes without losing exercise adherence. |  |  |  |  |  |  |  |  |  |  |  |
| 3.3.1 The optimal time for exercise is between 30 minutes and 2 hours after starting dialysis. |  |  |  |  |  |  |  |  |  |  |  |
| 3.3.2 The target exercise duration is 30–60 minutes (including warm-up and cool-down). At least 30 minutes of exercise should be completed, which can be done in one session or divided into multiple sessions based on the patient’s condition. |  |  |  |  |  |  |  |  |  |  |  |
| 3.3.3 The exercise program should last for at least 3 months. |  |  |  |  |  |  |  |  |  |  |  |
| 3.4.1 A single exercise session should include warm-up, exercise training, and cool-down. Warm-up: At least 5～10 minutes of low to moderate-intensity aerobic and muscle endurance exercises. Exercise phase: At least 20～60 minutes, including aerobic exercise, resistance exercise, and flexibility exercises. Cool-down: At least 5～10 minutes of low to moderate-intensity aerobic and muscle endurance exercises. |  |  |  |  |  |  |  |  |  |  |  |
| 3.4.2 During dialysis, a combination of aerobic and resistance exercises, resistance exercises alone, or aerobic exercises alone can improve dialysis adequacy. Probability ranking shows that a combination of aerobic and resistance exercises during dialysis has the best effect on improving dialysis adequacy. |  |  |  |  |  |  |  |  |  |  |  |
| 3.4.3 It is recommended that dialysis centers use motorized bicycles with adjustable resistance for routine aerobic and resistance exercises, as this method is simple and effective. Alternatively, a combination of aerobic, resistance, and flexibility exercises in a supine position can be performed during dialysis. |  |  |  |  |  |  |  |  |  |  |  |
| 3.4.4 Aerobic exercises during dialysis can include various movements of the non-arteriovenous fistula arm and both lower limbs, such as hand gripping, wrist rotation, elbow flexion and extension, and hip adduction, abduction, flexion, and extension. |  |  |  |  |  |  |  |  |  |  |  |
| 3.4.5 Resistance exercises during dialysis can include lifting dumbbells with the non-fistula arm, elastic band training, progressive ankle weight training, resistance band exercises, knee extension exercises, and progressive supine leg lifts. |  |  |  |  |  |  |  |  |  |  |  |
| 4.1.1 Monitoring should occur before, during, and after exercise, including vital signs and vascular access status. |  |  |  |  |  |  |  |  |  |  |  |
| 4.1.2 Strengthen monitoring during dialysis and assess exercise intensity. Closely observe the patient for any discomfort during exercise and identify potential causes. |  |  |  |  |  |  |  |  |  |  |  |
| 4.2.1 Persistent chest or back pain, palpitations, or chest tightness. |  |  |  |  |  |  |  |  |  |  |  |
| 4.2.2 Severe shortness of breath or difficulty speaking. |  |  |  |  |  |  |  |  |  |  |  |
| 4.2.3 Headache, dizziness, generalized weakness, visual disturbances, or profuse sweating. |  |  |  |  |  |  |  |  |  |  |  |
| 4.2.4 Severe arrhythmias. |  |  |  |  |  |  |  |  |  |  |  |
| 4.2.5 Exercise-related muscle cramps or joint pain. |  |  |  |  |  |  |  |  |  |  |  |
| 5.1.1 Use appropriate theories (e.g., Health Belief Model, Theory of Planned Behavior) to help patients adopt and adhere to exercise plans. |  |  |  |  |  |  |  |  |  |  |  |
| 5.2.1 Educate patients about exercise during dialysis, including indications, contraindications, benefits, risks, specific exercise protocols, precautions, and how to manage potential discomfort. |  |  |  |  |  |  |  |  |  |  |  |
| 5.2.2 Encourage patients to use diaries, informational manuals, or other visual tools to track exercise progress. Provide demonstration materials (e.g., manuals, videos) in advance. |  |  |  |  |  |  |  |  |  |  |  |
| 5.2.3 Train dialysis center staff on emergency response protocols. |  |  |  |  |  |  |  |  |  |  |  |

For Table 3, if you have any other comments or suggestions, please note them here:：___________________________________________________________________________________________________________________________________________________________________________________________________________________________________________________________________________________________________________________________________________________________________________________________________________

**Part III: Expert Familiarity and Basis of Judgment**

1. Your Basis for Judgment (The basis of judgment is divided into three levels: high, medium, and low. According to your actual situation, please check "√" under the corresponding level).

Expert Judgment Basis Survey Form

| Basis for Judgment Expert | Self-Assessment | | |
| --- | --- | --- | --- |
|  | High | Medium | Low |
| Theoretical Analysis | □ | □ | □ |
| Practical/Research Experience | □ | □ | □ |
| References(Domestic & International) | □ | □ | □ |
| Intuitive Judgment | □ | □ | □ |

1. Your Familiarity with the Consultation Content (The level of familiarity is divided into 5 grades, ranging from "Very Familiar" to "Not Very Clear". According to your actual situation, please check "√" in the corresponding column).

Familiarity Survey Form

| Familiarity Level | Very Familiar | Familiar | Basically Familiar | Slightly Familiar | Very Unfamiliar |
| --- | --- | --- | --- | --- | --- |
| Self-Assessment | □ | □ | □ | □ | □ |

This questionnaire ends here. Please check for any omissions.

Once again, we sincerely thank you for your valuable opinions and suggestions. We look forward to your continued guidance! Wishing you success in your work and good health!

**Appendix 2：**

**Development of an Exercise Intervention Program During Dialysis for Patients on Maintenance Hemodialysis Based on the Health Belief Model**

**Expert Consultation Questionnaire (Round 2)**

Dear Professors,

It is a great honor to invite you to serve as a consulting expert for the development of an exercise intervention protocol for maintenance hemodialysis patients. I am Cheng Xianjuan from Shengjing Hospital of China Medical University,, and my research topic is "Development and Application Effect Evaluation of an Exercise Intervention Protocol for Maintenance Hemodialysis Patients." Given your expertise in the relevant field, I am pleased to invite you once again to participate as an expert in the second round of consultations for this study.

Based on the results of the first round of consultations and after discussion within the research team, the following revisions have been made:

1. A secondary item titled "Monitoring Personnel" has been added, along with corresponding tertiary items.

2. Two tertiary items have been revised:

1）"Exercise Capacity Assessment: Measured by maximal oxygen uptake" has been changed to "Exercise Capacity Assessment: Since over 50% of maintenance hemodialysis patients may have difficulty completing maximal oxygen uptake testing, it is recommended to use simple physical function tests, such as the 6-minute walk test."

2） "Exercise frequency: 3 times per week" has been revised to "Exercise frequency: Adjusted according to the patient's dialysis schedule, 2–3 times per week."

3. Two tertiary items have been added:

1)Under the secondary item "Patient Preparation," the following has been included: "Ensure adequate dialysis. Clinical issues such as volume overload, metabolic acidosis, anemia, malnutrition, and electrolyte imbalances often increase the risks of exercise implementation."

2) Under the secondary item "Monitoring Content and Timing," the following has been added: "For diabetic patients, attention should be paid to the risk of hypoglycemia, and blood glucose levels should be monitored before and after exercise when necessary."

After these revisions, the second round of the consultation questionnaire now includes 5 primary items (Necessity of Exercise, Exercise Safety Assessment, Exercise Prescription, Exercise Monitoring, Exercise Support), 15 secondary items, and 43 tertiary items.

This expert consultation form consists of three parts:(1) Basic Information Survey for Experts;

(2) Expert Consultation Questionnaire on the Exercise Intervention Protocol for Maintenance Hemodialysis Patients;(3) Expert Self-Assessment Questionnaire on Familiarity (Cs) and Basis of Judgment (Ca).Please complete the forms and provide your feedback truthfully after reading the corresponding instructions. Additionally, your information will be used solely for research purposes. The research findings will be presented in academic papers, and your specific details will not be disclosed in any publication.

We sincerely hope you will provide valuable feedback. Your support is crucial to the successful progress of this study. Thank you for taking the time from your busy schedule. Wishing you all the best in your work!

Sincerely,

Cheng Xianjuan

Affiliation: Shengjing Hospital, China Medical University

Phone: 18940252396

Email: [1127200419@qq.com](mailto:1127200419@qq.com)

**Part I Expert Background Information Survey**

Instructions:To ensure the rigor of this study,please complete the following items based on your actual situation. Place a check mark (√) in the appropriate option. Thank you sincerely for your support of this research project!

| 1.Age：____ |
| --- |
| 2.Gender：Male Female |
| 3.Highest Degree Obtained：Bachelor's□ Master's or above |
| 4. Academic/Professional Title：Junior□ Intermediate□ Senior or above□ |
| 5.Years of Professional Experience: _____ years |
| 6.Research Field(s)：Nephrology□ Clinical Nursing□ Exercise Rehabilitation |

**Part II Expert Consultation Questionnaire on the Exercise Intervention Program**

Instructions for Completion:Please evaluate theimportance and operational feasibility of each item using the Likert 5-point scale. Scores range from 1 to 5 points, where 1 point indicates "Not Important/Not Feasible" and 5 points indicates "Very Important/Very Feasible".If you find the description of any indicator inaccurate or believe it should be deleted,please specify your reasons in the "Specific Revision Suggestions" column.If you believe there are relevant elements not yet considered in this study that should be added,please provide your additions in the "Additional Items" column.

Table 1 Consultation Form for Primary Indicators

| Primary Indicator | Item Importance | | | | | Item Operational Feasibility | | | | | Specific Revision Suggestions |
| --- | --- | --- | --- | --- | --- | --- | --- | --- | --- | --- | --- |
|  | 5  Very Important | 4  Fairly Important | 3  Moderately Important | 2  Slightly Important | 1  Not Important | 5  Very Strong | 4  Fairly Strong | 3  Moderate | 2  Slightly Strong | 1  Not Strong |  |
| 1.Necessity of Exercise |  |  |  |  |  |  |  |  |  |  |  |
| 2.Pre-Exercise Preparation |  |  |  |  |  |  |  |  |  |  |  |
| 3.Exercise Prescription |  |  |  |  |  |  |  |  |  |  |  |
| 4.Exercise Monitoring |  |  |  |  |  |  |  |  |  |  |  |
| 5.Exercise Support |  |  |  |  |  |  |  |  |  |  |  |

For Table 1, if you have any other comments or suggestions, please note them here: __________________________________________________________________________________

Table 2 Consultation Form for Second Indicators

| Second Indicator | Item Importance | | | | | Item Operational Feasibility | | | | | Specific Revision Suggestions |
| --- | --- | --- | --- | --- | --- | --- | --- | --- | --- | --- | --- |
|  | 5  Very Important | 4  Fairly Important | 3  Moderately Important | 2  Slightly Important | 1  Not Important | 5  Very Strong | 4  Fairly Strong | 3  Moderate | 2  Slightly Strong | 1  Not Strong |  |
| 1.1 Exercise Safety |  |  |  |  |  |  |  |  |  |  |  |
| 1.2 Exercise Benefits |  |  |  |  |  |  |  |  |  |  |  |
| 2.1 Preparations for Research Personnel |  |  |  |  |  |  |  |  |  |  |  |
| 2.2 Preparations for Patients |  |  |  |  |  |  |  |  |  |  |  |
| 2.3 Exercise Safety Assessment |  |  |  |  |  |  |  |  |  |  |  |
| 2.4 Exercise Contraindications |  |  |  |  |  |  |  |  |  |  |  |
| 3.1 Exercise Frequency |  |  |  |  |  |  |  |  |  |  |  |
| 3.2 Exercise Intensity |  |  |  |  |  |  |  |  |  |  |  |
| 3.3 Exercise Duration |  |  |  |  |  |  |  |  |  |  |  |
| 3.4 Type of Exercise |  |  |  |  |  |  |  |  |  |  |  |
| 4.1 Monitoring Personnel |  |  |  |  |  |  |  |  |  |  |  |
| 4.2 Timing and Content of Monitoring |  |  |  |  |  |  |  |  |  |  |  |
| 4.3 Indications for Exercise Termination |  |  |  |  |  |  |  |  |  |  |  |
| 5.1 Theoretical Support |  |  |  |  |  |  |  |  |  |  |  |
| 5.2 Social Support |  |  |  |  |  |  |  |  |  |  |  |

For Table 2, if you have any other comments or suggestions, please note them here: ___________________________________________________________________

Table 3 Consultation Form for Tertiary Indicators

| Tertiary Indicator | Item Importance | | | | | Item Operational Feasibility | | | | | Specific Revision Suggestions |
| --- | --- | --- | --- | --- | --- | --- | --- | --- | --- | --- | --- |
|  | 5  Very Important | 4  Fairly Important | 3  Moderately Important | 2  Slightly Important | 1  Not Important | 5  Very Strong | 4  Fairly Strong | 3  Moderate | 2  Slightly Strong | 1  Not Strong |  |
| 1.1.1 Exercise during dialysis is safe, and it is recommended that all treatment institutions provide exercise during dialysis to assist in treatment. |  |  |  |  |  |  |  |  |  |  |  |
| 1.2.1 Exercising for at least 60 minutes three times a week during dialysis can improve the survival rate of dialysis patients. |  |  |  |  |  |  |  |  |  |  |  |
| 1.2.2 Moderate-intensity resistance and aerobic exercise during dialysis can improve dialysis adequacy. |  |  |  |  |  |  |  |  |  |  |  |
| 1.2.3 Exercise during dialysis can significantly improve micro-inflammatory status, serum albumin levels, fatigue, and sleep conditions in dialysis patients. |  |  |  |  |  |  |  |  |  |  |  |
| 1.2.4 Exercise during dialysis can improve physical function, quality of life, cardiopulmonary function, and blood pressure levels in dialysis patients. |  |  |  |  |  |  |  |  |  |  |  |
| 1.2.5 Exercise during dialysis can alleviate psychological disorders such as depression and anxiety in patients. |  |  |  |  |  |  |  |  |  |  |  |
| 1.2.6 It is worth emphasizing that even a slight increase in physical activity levels may be beneficial. |  |  |  |  |  |  |  |  |  |  |  |
| 2.1.1 It is recommended that professional medical staff develop the exercise plan and implement it accordingly. |  |  |  |  |  |  |  |  |  |  |  |
| 2.1.2 Researchers should enhance their knowledge of relevant expertise, educate dialysis patients and their families about the implementation methods, safety monitoring, and outcome evaluation, and fully recognize its clinical benefits. |  |  |  |  |  |  |  |  |  |  |  |
| 2.2.1 Patients should be informed of the benefits and risks before exercise, and informed consent should be obtained from the patient/family, with a signed consent form. |  |  |  |  |  |  |  |  |  |  |  |
| 2.2.2 Ensure the patency of vascular access, as it is the lifeline for dialysis patients and a necessary condition for adequate dialysis. |  |  |  |  |  |  |  |  |  |  |  |
| 2.2.3 Ensure adequate dialysis. Clinical issues such as volume overload, metabolic acidosis, anemia, malnutrition, and electrolyte imbalances often increase the risks of exercise implementation. |  |  |  |  |  |  |  |  |  |  |  |
| 2.3.1 Clinical Condition Assessment: ① Medical history assessment: Symptoms, comorbidities (especially cardiovascular diseases, bone and joint abnormalities, etc.), medication history, lifestyle habits, exercise habits, family history, etc. ② Physical examination ③ Laboratory tests. |  |  |  |  |  |  |  |  |  |  |  |
| 2.3.2 Exercise Capacity Assessment: Since over 50% of MHD patients cannot complete maximal oxygen uptake (VO2max) testing, it is recommended to use simple physical function tests, such as the 6-minute walk test. |  |  |  |  |  |  |  |  |  |  |  |
| 2.4.1 Abnormal Blood Pressure: Severe hypertension (blood pressure exceeding 180/110 mmHg) or hypotension (blood pressure below 90/60 mmHg). |  |  |  |  |  |  |  |  |  |  |  |
| 2.4.2 Cardiopulmonary Diseases: Severe heart failure, arrhythmias, unstable angina, severe pericardial effusion, valvular stenosis, hypertrophic cardiomyopathy, aortic dissection, uncontrolled pulmonary hypertension (mean pulmonary artery pressure > 55 mmHg). |  |  |  |  |  |  |  |  |  |  |  |
| 2.4.3 Acute Clinical Events: Acute systemic inflammatory diseases or fever, acute phase of cardiovascular or cerebrovascular diseases, acute phase of traumatic injuries, etc. |  |  |  |  |  |  |  |  |  |  |  |
| 2.4.4 New-Onset Deep Vein Thrombosis Symptoms: Exercise should be postponed or stopped if symptoms such as abnormal redness, swelling, or pain in the calf occur. |  |  |  |  |  |  |  |  |  |  |  |
| 2.4.5 Patient Inability to Cooperate with Exercise: If the patient is unable to cooperate with the exercise program. |  |  |  |  |  |  |  |  |  |  |  |
| 3.1.1 Exercise frequency should be 2～3 times per week, based on the patient’s dialysis schedule. |  |  |  |  |  |  |  |  |  |  |  |
| 3.2.1 Start with low-intensity exercise and gradually progress to moderate-intensity exercise. |  |  |  |  |  |  |  |  |  |  |  |
| 3.2.2 It is recommended to use the Rating of Perceived Exertion (RPE) scale to determine exercise intensity. This scale is simple and practical. The exercise intensity is generally set at 12～14 points (the patient feels slightly tired but can still comfortably converse without significant strain). This ensures the patient achieves safe and effective exercise outcomes without losing exercise adherence. |  |  |  |  |  |  |  |  |  |  |  |
| 3.3.1 The optimal time for exercise is between 30 minutes and 2 hours after starting dialysis. |  |  |  |  |  |  |  |  |  |  |  |
| 3.3.2 The target exercise duration is 30–60 minutes (including warm-up and cool-down). At least 30 minutes of exercise should be completed, which can be done in one session or divided into multiple sessions based on the patient’s condition. |  |  |  |  |  |  |  |  |  |  |  |
| 3.3.3 The exercise program should last for at least 3 months. |  |  |  |  |  |  |  |  |  |  |  |
| 3.4.1 A single exercise session should include warm-up, exercise training, and cool-down. Warm-up: At least 5～10 minutes of low to moderate-intensity aerobic and muscle endurance exercises. Exercise phase: At least 20～60 minutes, including aerobic exercise, resistance exercise, and flexibility exercises. Cool-down: At least 5～10 minutes of low to moderate-intensity aerobic and muscle endurance exercises. |  |  |  |  |  |  |  |  |  |  |  |
| 3.4.2 During dialysis, a combination of aerobic and resistance exercises, resistance exercises alone, or aerobic exercises alone can improve dialysis adequacy. Probability ranking shows that a combination of aerobic and resistance exercises during dialysis has the best effect on improving dialysis adequacy. |  |  |  |  |  |  |  |  |  |  |  |
| 3.4.3 It is recommended that dialysis centers use motorized bicycles with adjustable resistance for routine aerobic and resistance exercises, as this method is simple and effective. Alternatively, a combination of aerobic, resistance, and flexibility exercises in a supine position can be performed during dialysis. |  |  |  |  |  |  |  |  |  |  |  |
| 3.4.4 Aerobic exercises during dialysis can include various movements of the non-arteriovenous fistula arm and both lower limbs, such as hand gripping, wrist rotation, elbow flexion and extension, and hip adduction, abduction, flexion, and extension. |  |  |  |  |  |  |  |  |  |  |  |
| 3.4.5 Resistance exercises during dialysis can include lifting dumbbells with the non-fistula arm, elastic band training, progressive ankle weight training, resistance band exercises, knee extension exercises, and progressive supine leg lifts. |  |  |  |  |  |  |  |  |  |  |  |
| 4.1.1 During the initial phase of exercise monitoring, nurses and doctors should jointly supervise. Once the patient masters the exercise method and stabilizes, nurses may monitor independently. Rehabilitation specialists should regularly participate in patient assessments and the development of subsequent exercise plans. |  |  |  |  |  |  |  |  |  |  |  |
| 4.2.1 Monitoring should occur before, during, and after exercise, including vital signs and vascular access status. |  |  |  |  |  |  |  |  |  |  |  |
| 4.2.2 Strengthen monitoring during dialysis and assess exercise intensity. Closely observe the patient for any discomfort during exercise and identify potential causes. |  |  |  |  |  |  |  |  |  |  |  |
| 4.2.3For diabetic patients, monitor blood glucose levels before and after exercise if necessary to mitigate hypoglycemia risks. |  |  |  |  |  |  |  |  |  |  |  |
| 4.3.1 Persistent chest or back pain, palpitations, or chest tightness. |  |  |  |  |  |  |  |  |  |  |  |
| 4.3.2 Severe shortness of breath or difficulty speaking. |  |  |  |  |  |  |  |  |  |  |  |
| 4.3.3 Headache, dizziness, generalized weakness, visual disturbances, or profuse sweating. |  |  |  |  |  |  |  |  |  |  |  |
| 4.3.4 Severe arrhythmias. |  |  |  |  |  |  |  |  |  |  |  |
| 4.3.5 Exercise-related muscle cramps or joint pain. |  |  |  |  |  |  |  |  |  |  |  |
| 5.1.1 Use appropriate theories (e.g., Health Belief Model, Theory of Planned Behavior) to help patients adopt and adhere to exercise plans. |  |  |  |  |  |  |  |  |  |  |  |
| 5.2.1 Educate patients about exercise during dialysis, including indications, contraindications, benefits, risks, specific exercise protocols, precautions, and how to manage potential discomfort. |  |  |  |  |  |  |  |  |  |  |  |
| 5.2.2 Encourage patients to use diaries, informational manuals, or other visual tools to track exercise progress. Provide demonstration materials (e.g., manuals, videos) in advance. |  |  |  |  |  |  |  |  |  |  |  |
| 5.2.3 Train dialysis center staff on emergency response protocols. |  |  |  |  |  |  |  |  |  |  |  |

For Table 3, if you have any other comments or suggestions, please note them here:：____________________________________________________________________________________________________________________________________________________________________________________________________________________________________________________________________________________________________________________________________________________________________________________________________________

**Part III: Expert Familiarity and Basis of Judgment**

1. Your Basis for Judgment (The basis of judgment is divided into three levels: high, medium, and low. According to your actual situation, please check "√" under the corresponding level).

Expert Judgment Basis Survey Form

| Basis for Judgment Expert | Self-Assessment | | |
| --- | --- | --- | --- |
|  | High | Medium | Low |
| Theoretical Analysis | □ | □ | □ |
| Practical/Research Experience | □ | □ | □ |
| References(Domestic & International) | □ | □ | □ |
| Intuitive Judgment | □ | □ | □ |

1. Your Familiarity with the Consultation Content (The level of familiarity is divided into 5 grades, ranging from "Very Familiar" to "Not Very Clear". According to your actual situation, please check "√" in the corresponding column).

Familiarity Survey Form

| Familiarity Level | Very Familiar | Familiar | Basically Familiar | Slightly Familiar | Very Unfamiliar |
| --- | --- | --- | --- | --- | --- |
| Self-Assessment | □ | □ | □ | □ | □ |

This questionnaire ends here. Please check for any omissions.

Once again, we sincerely thank you for your valuable opinions and suggestions. We look forward to your continued guidance! Wishing you success in your work and good health!

**Appendix 3：**

**Specific operational procedures for exercise intervention during dialysis based on the Health Belief Model**

I. Minimum Equipment and Environmental Requirements

1. Core Equipment (choose one or both):

· Simple Recumbent Exercise Bike: Capable of setting and adjusting resistance, equipped with timer/counter functions.

· Supine Exercise Equipment: Grip rings, sandbags for resistance training.

2. Monitoring Equipment: Bedside monitor (capable of continuous blood pressure, heart rate, and oxygen saturation monitoring).

3. Recording Tools: Exercise record form (paper or electronic version), pictorial card of the Rating of Perceived Exertion (RPE) scale.

4. Space Requirements: Sufficient space beside the dialysis bed to safely place the exercise bike without obstructing staff movement or emergency access.

II. Initial Patient Screening and Initiation Criteria

Before initiating exercise, the patient must meet all of the following conditions simultaneously:

· Clinically Stable Condition:

· Pre-dialysis blood pressure: Systolic ≥ 90mmHg and ≤ 180mmHg, Diastolic ≤ 110mmHg.

· Well-controlled dry weight, with no signs of acute fluid overload (e.g., dyspnea, pulmonary rales).

· Hemoglobin ≥ 90g/L, Serum albumin > 32g/L.

· No Exercise Contraindications: Absence of symptoms listed in the protocol such as acute clinical events, unstable cardiopulmonary disease, or newly developed deep vein thrombosis.

· Vascular Access Assessment: Well-functioning arteriovenous fistula, with no signs of redness, swelling, bleeding, or infection. The access site will be properly secured during exercise.

· Cognition and Willingness: Alert and oriented, able to understand and follow instructions, voluntarily participates, and provides signed informed consent.

III. Detailed Operating Procedure and Schedule (Single Session, Total Duration ≥ 30 minutes)

| Phase | Time | Activity Content | Specific Operations & Monitoring Points | Staff Responsibilities |
| --- | --- | --- | --- | --- |
| 0. Preparation | Before dialysis start | Assessment & Education | 1. Verify the patient meets the "Initial Initiation Criteria." 2. Educate the patient and family, explaining exercise safety and benefits. 3. Explain today's exercise plan, confirm patient comfort. 4. Check exercise equipment (bike resistance at zero, grip rings, sandbags intact). | Primary Nurse / Physician |
| 1. Warm-up | 5-10 minutes (Start 30 mins post-initiation) | Low-intensity Aerobic & Joint Mobility | 1. Equipment-free warm-up: Guide patient in slow, wide-range joint movements of the non-fistula limb (ankle pumps, knee flexion/extension, shoulder rotations). 2. Or, equipment warm-up: Set bike resistance to lowest, pedal at very slow speed (<30 RPM). Monitoring: Measure and record pre-exercise BP, HR. Ask patient about feelings. | Primary Nurse / Physician |
| 2.Main Exercise | 20-60 minutes (Can be completed in multiple bouts) | Mixed Exercise (preferred), Aerobic, or Resistance Exercise | Option A (Recumbent Bike):  • Intensity: Set resistance per reference table, maintain RPE 12-14 ("somewhat hard, but can talk").  • Method: Continuous or interval (e.g., 5 mins exercise, 1 min rest) cycling.  Option B (Supine Exercises):  • Movements: Non-fistula limb: grip exercises, elbow flexion/extension, limb raises/lowers, weighted limb raises/lowers, upper limb strength training (grip rings); Knee flexion/extension, leg raises/lowers, weighted leg raises/lowers, lower limb strength training (hip/lumbar raises with back extension).  • Intensity: Complete 10-15 reps/set per movement, repeat 2-3 sets, gradually increase intensity, 60-second rest between sets.  Monitoring (Throughout):  • Inquire RPE score every 5-10 minutes.  • Continuously observe monitor vitals and patient's complexion/expression.  • Immediately stop if any "Exercise Termination Criteria" from the protocol appear. | Primary Nurse / Physician |
| 3. Cool-down | 5-10 minutes | Low-intensity Movement & Stretching | 1. Reduce bike resistance to minimum, slow cycling for 5 mins. 2. Or perform slow limb swings. 3. Guide patient in static stretches for major muscle groups (hold each 15-30 sec), e.g., calf muscles.   Monitoring: Measure and record immediate post-exercise BP, HR. | 1. Primary Nurse |
| 4. Recording & Feedback | After exercise | Complete Record Form | 1. Briefly discuss with patient to understand their experience. 2. Fully complete the "Intradialytic Exercise Record Form." 3. Based on today's performance, plan next session's goals, and adjust the exercise plan monthly in consultation with the rehabilitation therapist. | Primary Nurse |

IV. Progression and Adjustment Criteria

1. Resistance Reference (Using the Exercise Bike as an Example)

| Resistance Level | RPE Score | Subjective Sensation Description | Applicable Phase |
| --- | --- | --- | --- |
| Level 1 (Low) | 9-11 (Very Light) | Almost no effort, steady breathing | Initial/Recovery Phase |
| Level 2 (Medium) | 12-14 (Somewhat Hard) | Feels slightly tired, but able to hold a full conversation | Maintenance Phase |
| Level 3 (High) | 15-16 (Hard) | Clearly feels tired, speech requires pauses | Advanced Phase seeks increased challenge |

2. Summary Table of Initial and Progression Standards

| Assessment Dimension | Initial Start Standard | First Stage Progression Standard | Second Stage Progression Standard (Goal) |
| --- | --- | --- | --- |
| Single Session Duration | 15-20 minutes (including warm-up/cool-down) | Increase to 25-40 minutes | Stabilize at 45-60 minutes |
| Exercise Frequency | Conducted during 1-2 dialysis sessions per week | Conducted during 2-3 dialysis sessions per week | Conducted during every dialysis session |
| Exercise Intensity (RPE) | 9-11 points | Stabilize at 12-14 points | Able to complete target duration at 14 points |
| Resistance Level | Level 1 (Low) | Level 2 (Medium) | Level 2 (Medium) |
| Program Duration | Continuously for at least 2 weeks | Fatigue recovers quickly after completing target duration,Continuously for at least 3 months | Long-term adherence |

Progression Method: Priority should be given to increasing exercise duration (e.g., from 20 to 30 minutes), followed by increasing exercise frequency (from 1-2 sessions per week to 2-3 sessions per week of dialysis treatment), and finally, considering an increase in resistance intensity (moving up one level).

V. Exercise Termination and Safety Emergency Procedures

1. Immediate Exercise Termination Criteria: Stop immediately if any termination criterion specified in the exercise intervention protocol occurs.

2. Emergency Procedure:

· Stop exercise, assist patient to rest quietly.

· Assess vital signs, notify physician.

· Manage symptoms according to protocol: e.g., for hypotension, place in Trendelenburg position, administer fluid bolus.

· Document event: Record the adverse event and management process in detail on the record form and in nursing documentation.

VI. Recording Template

Intradialytic Exercise Record Form

| Date: | Patient Name: | Dialysis Duration: _______h_______min |
| --- | --- | --- |
| Pre-exercise Assessment |  |  |
| Exercise Prescription | Type: □ Exercise Bike □Supine Exercises Other____ | Target Duration: _____ min |
| Exercise Execution | Start Time: ________ | Actual Duration: _____ min |
| Post-exercise Assessment | BP：_______/_______ mmHg | HR: _______ bpm |
| Adverse Events: | Remarks/Next Session Plan: | Nurse Signature: |

VII. Staff Workflow and Responsibilities

· Dialysis Center Director/Head Nurse: Responsible for program promotion, resource (equipment, training) support, and quality supervision.

· Primary Nurse (Core Implementer):

· Pre-treatment: Screen patients, complete initiation assessment, develop individualized exercise prescriptions.

· During treatment: Execute exercise monitoring according to procedure, ensure safety, and promptly manage abnormalities.

· Post-treatment: Complete documentation, provide feedback and encouragement to patients.

· Rehabilitation Therapist (Technical Support):

· Conduct regular (e.g., monthly) reassessment of patient exercise capacity (e.g., 6-Minute Walk Test).

· Update exercise prescriptions jointly with nurses based on assessment results.

· Provide advanced training and guidance on complex cases to nurses.

· Attending Physician:

· Review patient eligibility and manage exercise-related adverse events.

· Collaborate on adjusting exercise prescriptions from a medical perspective (e.g., due to changes in condition).
